# Advanced microfluidic devices for fabricating multi‐structural hydrogel microsphere

**DOI:** 10.1002/EXP.20210036

**Published:** 2021-12-17

**Authors:** Zehao Chen, Zhendong Lv, Zhen Zhang, David A. Weitz, Hongbo Zhang, Yuhui Zhang, Wenguo Cui

**Affiliations:** ^1^ School of Mechatronic Engineering and Automation Shanghai University Shanghai P. R. China; ^2^ Department of Orthopaedics Shanghai Key Laboratory for Prevention and Treatment of Bone and Joint Diseases Shanghai Institute of Traumatology and Orthopaedics Ruijin Hospital Shanghai Jiao Tong University School of Medicine Shanghai P. R. China; ^3^ Department of Spine Surgery Renji Hospital Shanghai Jiao Tong University School of Medicine Shanghai P. R. China; ^4^ Department of Physics and Harvard John A. Paulson School of Engineering and Applied Sciences Harvard University Cambridge Massachusetts USA; ^5^ Pharmaceutical Sciences Laboratory Åbo Akademi University and Turku Bioscience Centre University of Turku and Åbo Akademi University Turku Finland

**Keywords:** biomaterials, hydrogel microspheres, microfluidic devices, microfluidics

## Abstract

Hydrogel microspheres are a novel functional material, arousing much attention in various fields. Microfluidics, a technology that controls and manipulates fluids at the micron scale, has emerged as a promising method for fabricating hydrogel microspheres due to its ability to generate uniform microspheres with controlled geometry. With the development of microfluidic devices, more complicated hydrogel microspheres with multiple structures can be constructed. This review presents an overview of advances in microfluidics for designing and engineering hydrogel microspheres. It starts with an introduction to the features of hydrogel microspheres and microfluidic techniques, followed by a discussion of material selection for fabricating microfluidic devices. Then the progress of microfluidic devices for single‐component and composite hydrogel microspheres is described, and the method for optimizing microfluidic devices is also given. Finally, this review discusses the key research directions and applications of microfluidics for hydrogel microsphere in the future.

## Graphical abstract

The review summarizes the features of hydrogel microspheres and microfluidic technique, followed by the discussion of material selection in fabricating microfluidic device. Then the progress of microfluidic devices for both single‐component and composite hydrogel microspheres is described. Finally, this review discusses the key research direction and application of microfluidics for hydrogel microsphere in the future.

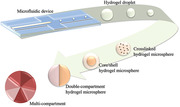

## 1 INTRODUCTION

Hydrogels, a class of polymeric materials with 3‐dimensional network structure, have been widely used in various fields, such as biomedicine, sensors, and soft robotics.^[^ ^1^, ^2^, ^3^ ^]^ Due to their tunable properties and abilities to mimic the natural extracellular matrix (ECM), hydrogels are particularly suitable for tissue engineering and regeneration medicine. Hydrogels are prepared by crosslinking polymer chains through diverse mechanisms, including physical self‐assembly, chemical crosslinking, and ionic interactions.^[^ ^4^, ^5^, ^6^ ^]^ However, conventional biomedical hydrogels´ implantation always requires surgical procedures at the target tissue, which increase the risk of infections and treatment cost. Currently, diverse injectable hydrogels such as thermosensitive and photo‐crosslinking hydrogels have been developed to implement minimally invasive injection therapy.^[^ ^7^, ^8^ ^]^ Those hydrogels can be delivered precisely to the objective regions through small needles and then cured in situ; however, bulk hydrogels still suffer from many shortcomings. For instance, photo‐crosslinking hydrogels are formed through exposure to UV or visible light, and their low penetration depth may result in the heterogeneity of the hydrogel in situ.^[^ ^9^ ^]^ Thermosensitive hydrogels, on the other hand, are formed through reversible physical crosslinking, so they exhibit poor mechanical properties and tend to collapse in the body.^[^ ^10^ ^]^

Hydrogel microspheres, also named as microgels, have been prepared to overcome the disadvantages of bulk hydrogels and have shown great potential in various fields. Hydrogel microspheres reserve most of the original properties of bulk hydrogels, while they can be completely crosslinked outside the body as uniformed micrometer sizes microspheres, thus can still be injected with minimal invasive injection. This effectively solves the problems of both injectability and the insufficient crosslinking of conventional hydrogels. Another advantage of microgels is their enlarged specific surface area that leads to enhanced adsorption capacity, making them promising agents in drug loading and contaminant removal.^[^ ^11^ ^]^ Moreover, microspheres are able to couple with bulk hydrogels. For instance, engineered granular hydrogels can be built by interactions among microgels to build up more complex structures to meet the demands of tissue engineering, such as controlled drug delivery and degradation rate.^[^ ^12^ ^]^ By contrast, microgels can be embedded into bulk hydrogels to build a composite hydrogel system, which exhibits highly tunable properties with prolonged drug release profiles.^[^ ^13^ ^]^

Various techniques have been used in the fabrication of hydrogel microspheres in recent years. In most cases, the preparation of hydrogel microspheres consists of two steps: (i) The generation of pre‐crosslinking hydrogel droplets. (ii) Solidify the droplets via different crosslinking strategies. Approaches of hydrogel droplet formation include batch emulsions, lithography, electrohydrodynamic spraying, mechanical fragmentation, and microfluidics.^[^ ^14^ ^]^ However, most of these fabrication approaches face some drawbacks. For example, most of these manufacturing methods can only generate polydisperse microspheres, which is particularly unfavorable when hydrogel microspheres are used as drug carriers to precisely control the drug release. Although photolithography is capable of generating microspheres of uniform size, it requires expensive equipment and does not realize mass production.

Among these techniques, microfluidics is a promising approach that can meet several requirements for microgel fabrication. It can control and manipulate thee fluids in micro‐meter size channels.^[^ ^15^ ^]^ As a subclass of microfluidic technology, droplet microfluidics based on fluid immiscibility mechanism is particularly suitable for producing hydrogel microspheres. Typically, two or multiple immiscible fluids are injected into the microfluidic device and then meet at the junction to generate diverse droplet geometries. As one kind of water‐soluble polymer, hydrogels can be easily prepared into an aqueous phase for microfluidic fabrication. In some cases, hydrophilic drugs and cell suspension are mixed into the hydrogel solution for the one‐step formation of microfluidic microsphere vehicles. In the process of forming hydrogel microspheres, microfluidic technique is able to break up a continuous sol fluid into dispersed hydrogel droplets. Compared with other manufacturing methods, microfluidics enables the generation of highly monodisperse microspheres at a moderate production rate, making it possible to achieve the controlled release of the encapsulated payloads. At the same time, the size and geometry of the microspheres can be accurately controlled through the adjustment of microfluid parameters, such as flow velocity and junction geometry. So microfluidic hydrogel microspheres can be optimized and tailored according to the characteristics of tissues and organs. Another merit is its comparable low requirement for equipment, which can dramatically reduce the production cost.

**FIGURE 1:**
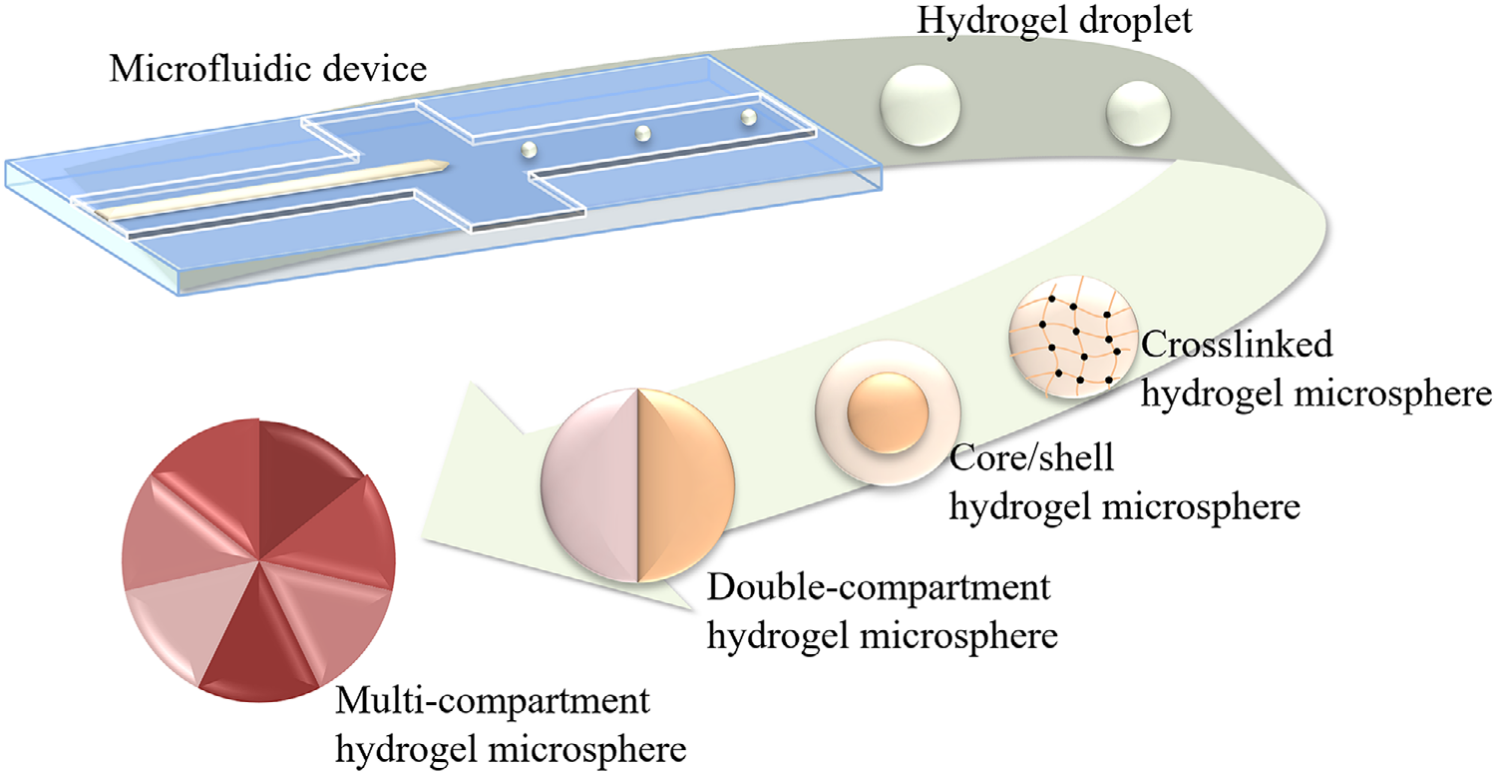
Hydrogel microspheres with diverse structure produced by microfluidic technology

The development and research of microfluidic devices are critical to hydrogel microspheres, and both the microchannel design and materials used for microfluidic device are the key factors in determining the properties of the microspheres. Classical droplet microfluidics has been used to fabricate hydrogel microspheres; however, it can only produce single‐component microspheres. In addition, various modifications have been made to the conventional microfluidic devices to generate microspheres with more complicated structures (Figure 1).

In this paper, the research process of microfluidic devices for hydrogel microspheres preparation is summarized. First, the main materials used to fabricate microfluidic devices and their corresponding manufacturing techniques are introduced. Next, the recent advances in microfluidic devices for preparing diverse hydrogel microspheres, including single‐component microspheres and composite microspheres are described. Optimization strategies to improve device performance are also discussed. Finally, conclusions and prospects of the future development of microfluidic devices for hydrogel microspheres are presented.

## 2 MATERIALS FOR MICROFLUIDIC DEVICE

Material selection is the first step in microfluidic device fabrication. To produce hydrogel microspheres, microchannels must be patterned on a material to form droplet generator geometry via appropriate fabrication strategies. Various materials have been utilized to fabricate microfluidic devices and exhibit diverse characteristics, which not only determine the manufacturing process of forming microchannels but further affect the flow and function of the microfluids. Thus, the selection of materials plays a vital role in the hydrogel microsphere formation. In this section, we introduce typical materials for microfluidic devices, including glass, silicon, polydimethylsiloxanes (PDMS), thermoplastics, and hydrogels. The fabrication processes are also represented respectively. Besides, some novel materials for special purposes are also presented in this section. The comparison of materials for microfluidics is shown in Table 1.

**TABLE 1:** Materials used in fabricating microfluidic devices

**Materials**	**Transparency**	**Mechanical strength**	**Fabricating process**	**Chemical resistance**	**Design flexibility**
Silicon	Yes	High	Etching and mechanical processing	High	Moderate
Glass	Yes	High	Etching, mechanical processing, and modular assembly	High	Moderate
PDMS	No	Low	Soft lithography	Moderate	Moderate
Thermo plastics	Yes	High	Hot embossing, laser ablation, etching, mechanical processing, and 3D printing	Moderate	High
Hydrogel	Yes	Low	Lithography, 3D printing, and sacrificial template	Low	High
Paper	No	Low	Physical or chemical deposition	Low	Moderate
Photo‐sensitive resin	No	Low	Soft lithography and 3D printing	High	High

### 2.1 Glass and silicon

Glass possesses a high degree of optical transparency, chemical resistance as well as excellent biocompatibility. To form the microchannels on a glass substrate, both chemical and mechanical processes are applied, such as wet or dry etching and mechanical cutting.^[^ ^16^ ^]^ Chemical processing aims to remove the materials through chemical reactions to create microchannels with precise profiles, while mechanical processing involves direct cutting on the glass. However, most of these processing methods are costly and require micromachining equipments.

Glass capillary emerges as a promising approach for manufacturing microfluidic devices.^[^ ^17^ ^]^ Unlike the manufacturing methods that require complex processing on glass substrates to form microchannels, glass capillaries themselves can be used as microchannels, thus microfluidic devices can be prepared via this module assembly strategy. The greatest advantage of glass capillary‐based devices is the significant reduction of production costs as well as the simplicity in device manufacturing. Various microspheres with different structures have been successfully prepared through diverse capillary assembly schemes.^[^ ^18^ ^]^

Despite the wide range of applications, some drawbacks exist for glass capillary‐based devices. For instance, as the geometry of the glass capillary is an invariable parameter, the modular assembly of glass capillaries does not allow the construction of precise and complicated microfluidic system. In most cases, the glass capillary with a top end is called injection capillary, which is inserted into another capillary called collection capillary to build a simple‐emulsion system. Since this insertion process is done manually, reproducibility of device fabrication remains a huge challenge.^[^ ^19^ ^]^ Besides, the minimum droplet sizes that the devices can produce are restricted, which is always in correlation to the minimum featured sizes for the microchannels.^[^ ^20^ ^]^ Therefore, glass capillary‐based devices are limited in generating microspheres at smaller sizes.

Silicon has been the prime material in the field of microelectromechanical system and micro manufacturing for many years due to its semiconducting property, ready availability and flexibility to design.^[^ ^21^ ^]^ Similar to glass, the chemical inertness of silicon allows for the reactions of a wide range of solvents, including organic solvents. Meanwhile, the silanol group of silicon makes it prone to have chemical modifications.^[^ ^22^ ^]^ The conventional method for fabricating silicon microfluidic devices is etching. Typically, the pattern of the microchannels is etched onto a silicon substrate via wet or dry etching, and then the substrate is sealed with an anodically bonded glass cover. Silicon devices generally exhibit high productivity and good repeatability owing to the high manufacturing accuracy of the etching process. However, as a non‐transparent material, silicon devices cannot be used for direct fluid imaging and measurement. As silicon devices are always processed via micromachining methods, there are high demands for working facilities and labor training, which bring high costs. It is also challenging to control the cross‐section profile of channels during the etching process. In most cases, photolithographic techniques can only be used to form the channels with rectangular cross‐sections.^[^ ^23^ ^]^ This channel geometry exhibits non‐uniform stress distribution, such as varying shear stress across the bottom of the channel.^[^ ^24^ ^]^ Although efforts have been made to produce channels with various cross‐sectional shapes,^[^ ^25^ ^]^ multiple photolithography processes are needed, which will significantly increase the manufacturing cost. Besides, most of these novel geometries are based on 2D planer, such as parallelogram, rhombus, pentagon, and hexagon.^[^ ^26^ ^]^ How to form circular channels on the silicon substrate is still an unresolved challenge.

### 2.2 PDMS

PDMS, a kind of thermoset material, is an excellent candidate material for microfluidic devices, due to its low cost, optically transparency, biological inertness, and prototyping capability.^[^ ^27^ ^]^ Soft lithography is the most common method for fabricating a PDMS device since it allows for both rapid and mass production.^[^ ^28^ ^]^ Conventionally, a photomask with channel patterns is firstly designed, and then the pattern is transferred to a photoresist layer to obtain the mold. Then liquid PDMS pre‐polymer is cast on the mold and cured through thermal treatment to form channel structure, followed by a sealing processing where another sheet material is bonded to the PDMS substrate automatically via oxygen‐plasma oxidation. As with silicon‐based devices, the mold prepared by soft lithography is limited by a single‐level pattern. In most cases, microchannels exhibit rectangular cross‐sectional geometries, with the same height in all directions.^[^ ^29^ ^]^ Alternatively, 3D printing strategy has been proposed to produce the mold, with the merit of more flexibility in the channel design with higher precision.^[^ ^30^ ^]^ Compared with the capillary assembly, PDMS devices exhibit excellent reproducibility with highly similar geometry using soft lithography and 3D printing. It is well known that these advanced manufacturing processes are cockamamie and costly. Considering the high demands of micromachining technology for experimental conditions, Xi et al.^[^ ^31^ ^]^ used microtubes to achieve low‐cost assembly of PDMS microfluidic. The microchannel was produced by passing a heated metal filament through PDMS precursor to generate a PDMS coating around the filament. After full solidification, the microtube was obtained by separating the wire and PDMS coated on its surface and could be further assembled into microfluidic devices.

Although PDMS is the most popular material for fabricating microfluidic devices, some of its characteristics limit its applications. Primarily, as a class of elastomeric material, PDMS possesses poor mechanical property, which may lead to the deformation of devices under high stress.^[^ ^32^ ^]^ Meanwhile, the hydrophobicity of PDMS is unexpected, limiting its use in systems with continuous aqueous phases. Also, the porous nature of PDMS may lead to the contamination of channels when devices are infused with fluids containing protein molecules, as PDMS is capable of absorbing these molecules.^[^ ^33^ ^]^ Therefore, extra modifications are always needed to improve the surface properties of PDMS microchannels. PDMS devices are also restricted with a certain scope of solvents since some specific solvents are able to react with them. For example, organic solvents can result in the swelling of PDMS and further influence the microfluidic devices.^[^ ^34^ ^]^

### 2.3 Thermoplastics

Thermoplastic materials are competitive substitutes due to their potential for mass manufacture.^[^ ^35^ ^]^ The common thermoplastics include polymethylmethacrylat (PMMA), polyvinylchlorid, and poly tetrafluoroethylene. Among them, PMMA is one kind of biocompatible material, with optical transparency as well as low water absorption and excellent water resistance.^[^ ^36^ ^]^ Thermoplastic device can be fabricated using conventional micromachining technologies, such as etching and milling processing.^[^ ^37^, ^38^ ^]^ Moreover, its thermosensitive nature allows for highly flexible designs. Thermoplastics will melt when heated and then gradually solidify as the temperature decreases. Hot embossing is a common method for tailoring thermoplastic materials.^[^ ^39^ ^]^ As one kind of molding replication method, hot embossing can also be divided into two processes: (i) Design a molding mask with microchannel patterns. (ii) Transfer the pattern to the thermoplastics and bond a layer to the thermoplastic substrate to complete the sealing. Compared with the soft lithography, hot embossing is characterized by relatively low cost and low requirements for equipments. Laser ablation is another fabricating method, using pulsing laser to cut row thermoplastic materials.^[^ ^40^ ^]^ In addition to these subtractive manufacturing methods, additive manufacturing can also be used to fabricate thermoplastic devices. For instance, a PMMA‐based microfluidic chip has been successfully built via fused deposition modeling technology.^[^ ^41^ ^]^ Moreover, sustainability is a unique merit of thermoplastics since they possess the ability to be remolded or recycled via heating, whereas other materials are readily disposable once they are processed.^[^ ^42^ ^]^

However, the chemical resistance of thermoplastic materials is not as good as glass and silicon. Under some circumstances, when devices are in contact with strong polar solvents and acids, the microchannel will be deformed.^[^ ^22^ ^]^ It is also challenging to fabricate thermoplastic devices at high temperature. The stability of the device during the bonding process is another issue that cannot be neglected, as most thermoplastic devices are deformed by significant changes in Young's modulus during the bonding process.^[^ ^43^ ^]^

### 2.4 Hydrogels

Hydrogel‐based microfluidic devices have also been developed recently due to their superior biocompatibility, which is particularly beneficial for fluids containing cells. Hydrogels are capable of forming channel structures through different strategies, including 3D printing, photolithography, and sacrificial template methods.^[^ ^44^, ^45^ ^]^ In addition, hydrogel materials exhibit intrinsic hydrophilic surfaces and do not require extra complex processing to functionalize like PDMS. However, it must be noted that hydrogel‐based devices also face the drawbacks of lower mechanical properties compared with devices made from traditional rigid materials. Due to these properties, the microchannels of hydrogel‐based devices tend to deform or even collapse under large stress, restricting their applications. Moreover, most hydrogel materials exhibited shortcomings of water absorption and swelling performance,^[^ ^46^ ^]^ thus maintaining the initial morphology and channel geometry of hydrogel‐based devices after inlet phase filling remains a big problem. Fortunately, some efforts have been made to overcome these limitations. For example, chemical modification of hydrogels is an effective tactic to enhance their mechanical strengths and stabilities. Chong et al.^[^ ^47^ ^]^ used di‐acrylated Pluronic F127 (F127‐DA), a photo‐crosslinked hydrogel material presenting non‐swelling property, to fabricate microfluidic device. Devices made from F127 exhibited the capability of maintaining initial mechanical strength and channel morphology when exposed to aqueous solution environment at room temperature. In spite of the merits of chemical modifications, the introduction of chemical groups will inevitably reduce the biological activity of hydrogels. To solve this issue, Ma et al.^[^ ^48^ ^]^ utilized a gelatin–riboflavin mixture to manufacture microfluidic devices. Gelatin could be photo‐crosslinked with riboflavin under blue‐light illumination and remained stable throughout the process. Since the hydrogel was free from chemical functionalization, the good biocompatibility of gelatin was preserved. On the other hand, although the shape change of hydrogel is unexpected in most cases, interestingly, sometimes the deformation of hydrogel can be utilized. A tunable microfluidic chip was produced by integrating pH‐sensitive hydrogel into PDMS microchannels.^[^ ^49^ ^]^ The pH‐sensitive property of the hydrogels allowed switching between the swelling state and the shrinking state when environmental pH value changed, exhibiting greater flexibility in manipulating microfluids.

### 2.5 Other materials

Currently, more and more materials are being used for the preparation of microfluidic devices and show great potential. The application of photosensitive resins in microfluidics has received widespread attention. Soft lithography is a common strategy for manufacturing photosensitive resin devices.^[^ ^50^ ^]^ In addition, the curing ability of photosensitive resins under digital light exposure allows 3D printing, exhibiting greater design flexibility. The use of photosensitive resins to construct microfluidic platforms by stereolithography has been proposed.^[^ ^51^ ^]^ Compared with photo‐crosslinking hydrogels, photosensitive resins show lower water swelling properties and remain stable in an aqueous environment for long period of time. However, photosensitive resins face the defect of non‐transparency and low mechanical property. 3D printed resin‐based microfluidics also cannot match the fabrication resolution of soft lithography,^[^ ^52^ ^]^ restricting its application in micro‐manufacturing.

Paper, a naturally derived material, is gradually being used in manufacturing microfluidic devices.^[^ ^53^ ^]^ Paper possesses high porosity, microstructure, hydrophily, and the ability to be functionalized to change its surface properties.^[^ ^54^ ^]^ Conventionally, to form a channel pattern on the paper substrate, some certain areas are modified to be hydrophobic while other areas remain hydrophilic, so that the hydrophilic region of the paper substrate can be used as channels. The aqueous phase is then allowed to wick along the hydrophilic areas due to capillary forces.^[^ ^55^ ^]^ Wax drawing is a common modification approach, which is low in cost and does not require sophisticated equipment. The main drawback of this approach is its low manufacturing accuracy. Thus, lithography technique has been applied to form the channel pattern on the paper substrate; however, it dramatically increases the fabrication cost.^[^ ^56^ ^]^ Another issue is that the hydrophilic property of the paper substrate makes it challenging to form an enclosed microfluidic channel,^[^ ^57^ ^]^ thus the majority of the paper‐based microfluidic devices are with open‐channel structure, which will interfere with the stability of water‐in‐oil (W/O) emulsion. Another aspect that limits the application of paper‐based devices is their poor mechanical properties, especially when the devices are under a wet state.

### 2.6 Selection principle of materials for microfluidics

Each of the above materials has pros and cons when used in microfluidic device manufacturing. In the case of microgel fabrication, the choice of materials for microfluidic devices depends on the type of microgel and its crosslinking method. For most photo‐ and physically crosslinked microgels, there exists no specific requirements for material selection. The machining way of the material may be the main concern, as it requires complex instruments for microfabrication. For instance, sophisticated photolithography equipment and 3D printers are essential for manufacturing PDMS and thermoplastic microfluidic devices. However, when organic solutions are involved in the liquid phase, glass and silicon devices are considered to be a better solution.^[^ ^58^, ^59^ ^]^ Besides, for the convenience of the real‐time observation of droplet generation, optically transparent materials are preferred. Therefore, assembly‐based capillary devices are probably the first option for most laboratories owing to their low barrier to entry.

In some specific applications, hydrogel microspheres are used as cell carriers. Since cells always need to be suspended in a hydrogel solution to form cell‐laden droplets, the biological compatibility of the material also matters. Although most materials for microfluidics exhibit moderate to high biocompatibility, in some studies, photosensitive resins have been reported to affect cellular behavior because the unreacted components during the polymerization of photosensitive resins exhibit cytotoxic properties.^[^ ^60^ ^]^ It is recommended that inorganic materials should be given priority to retain cellular activity.

## 3 MICROFLUIDIC DEVICES FOR SINGLE‐COMPONENT HYDROGEL MICROSPHERE

Primary microfluidic devices are used for single‐component hydrogel microsphere. In addition to material selection, another critical aspect of microfluidic devices is the design of the microchannel junctions. The geometrical factors of microchannel determine the flow behavior and droplet generation in the microchannels. Therefore, understanding the microfluid dynamics and droplet generation mechanisms is of great significance for designing microfluidic devices. There are several forces involved in the process of droplet generation, including viscous force, inertial force, and interfacial tension. As the size of channels in microfluidic platform is miniaturized to the micro‐scale, the inertial force becomes very small and can be neglected at normal flow velocities. Reynold number is proposed to describe the inertial forces. The microfluid under low Reynold number has laminar flow characteristics.^[^ ^61^ ^]^ Laminar flow is defined as a fluid in which velocity, pressure, and other flow properties maintain constant. In microfluidic systems, droplets are formed when two immiscible liquids meet at the junction. Thus, viscous force and interfacial force dominate in the behavior of microfluids. Two parameters have been used to indicate these forces: Capillary number ($N_{c}$) and Weber number ($N_{w}$). The capillary number ($N_{c}$) can be described with the Equation (1):

(1) $$N_{c}=\frac{\mu_{c}Q_{c}}{\sigma }\;$$

where $Q_{c}$ indicates the flow rate of inner phase and $\mu_{c}$ represents the dynamic viscosity of the continuous phase. σ is the interfacial tension coefficient between continuous and disperse phases.

Weber number ($N_{w}$) is defined in Equation (2):

(2) $$N_{w}=\frac{\rho \nu^{2}l}{\sigma }$$

where *ρ* is the fluid density, and σ indicates the interfacial tension between two liquids. ν and l denote the characteristic velocity and the characteristic length scale, respectively.

In short, capillary number reflects the relations between viscous forces and surface tensions, while Weber number shows the interactions of inertial and surface tension forces. The droplet regime is mainly divided into two categories: dripping and jetting. As the flow velocities of two phases are at a moderate level ($N_{c}$ value is low), a dripping regime occurs, in which droplets are formed at the junction. When the flow rate is very high, the disperse phase will not break up immediately at the junction, but form an extended jet before the droplets are generated at downstream of the channel. To prepare single‐component hydrogel microspheres, a single emulsion system is in need, which includes a dispersed phase and a continuous phase. For the formation of hydrogel droplets, the dispersed phase is always comprised of pre‐crosslinking hydrogel solution, while the oil is used as continuous phase. The low $N_{c}$ and $N_{w}$ lead to the formation of the dripping of the water phase.^[^ ^62^ ^]^ Moreover, the structure of the microchannel plays a vital role in droplet generation, which further influences the morphology of the hydrogel microspheres. The three basic geometric designs of microchannels are flow‐focusing, cross flow, and co‐flow. And the comparison of hydrogel microspheres prepared from these geometries is shown in Table 2. In this section, we present the droplet formation mechanisms of these basic channel designs and their applications in preparing single‐component hydrogel microspheres.

**TABLE 2:** Comparison of hydrogel microspheres produced by classical microfluidic geometry

**Droplet formation geometry**	**Type of hydrogel**	**Mean size**	**CV**
Flow focusing	GelMA^[^ ^64^ ^]^	100 μm	/
	Alginate^[^ ^65^ ^]^	5–20 μm	3.7–16.0
	PAAm^[^ ^66^ ^]^	363–571 μm	/
	PEG‐4MAL^[^ ^67^ ^]^	64–105 μm	6.7–13.7
	Dextran^[^ ^68^ ^]^	150–1000 μm	6.7–8.4
	Alginate^[^ ^69^ ^]^	90.4 μm	3.3
Cross flow	4‐arm PEG^[^ ^70^ ^]^	355 μm	2.5
	Chitosan^[^ ^71^ ^]^	19.2–31.6 μm	7.3–9.9
	pNIPAm^[^ ^72^ ^]^	81 μm	2.8
	PEG^[^ ^73^ ^]^	50–90 μm	1–2
	PF, GelMA and PEGDA^[^ ^74^ ^]^	700–1000 μm	2.6–6.2
Coflow	GelMA/chitosan^[^ ^77^ ^]^	54–350 μm	6.0–9.3
	GelMA^[^ ^78^ ^]^	200 μm	15
	HAMA/HepMA^[^ ^79^ ^]^	256.6 μm	2.4
	PEGDA^[^ ^80^ ^]^	50–200 μm	/
	Alginate^[^ ^81^ ^]^	150–900 μm	/

### 3.1 Flow‐focusing device

**FIGURE 2:**
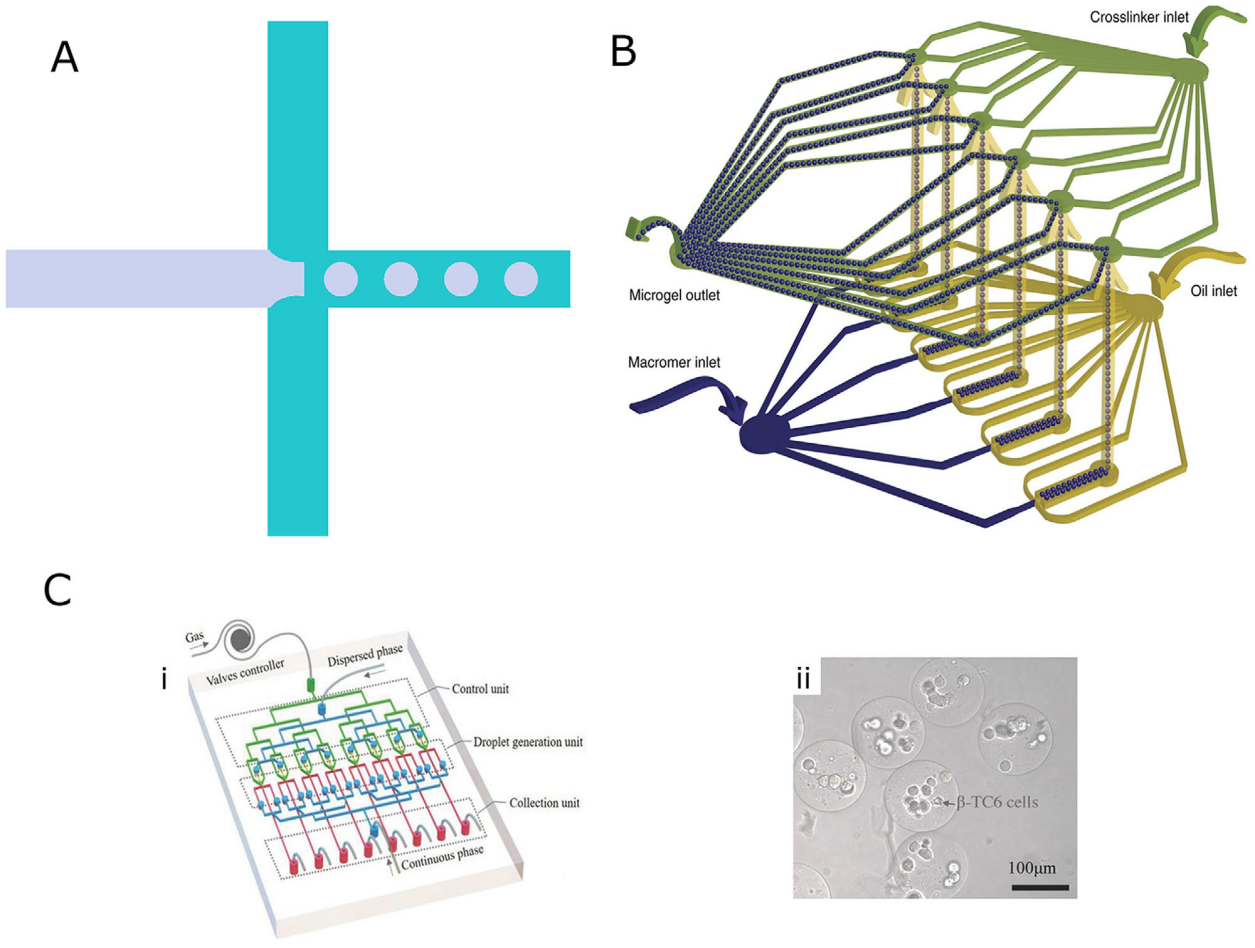
(A) Flow focusing mechanism. (B) Schematic of two‐layer microfluidic device with parallel flow focusing geometry. Reproduced with permission.^[^ ^67^ ^]^ Copyright 2018, Springer Nature. (C): (i) Schematic illustration of pneumatic valve‐assisted microfluidic system for generating W/W hydrogel microsphere. (ii) Bright‐field image of cells loaded on hydrogel microspheres after encapsulating 24 h. Reproduced with permission.^[^ ^69^ ^]^ Copyright 2018, John Wiley & Sons

In flow focusing, the disperse phase encounters the continuous phase at a crossing junction, squeezed by the continuous phase from the top and bottom (Figure 2A). Droplets are created because of the hydrodynamic focusing exerted by the continuous phase. In the flow‐focusing geometry, when the flow velocities of the disperse and continuous phases are not very high, a dripping state will occur, and droplets generated in this state typically exhibit a high monodispersity. On the other hand, with the increase of flow rate, the rising in the $N_{c}$ value will lead to a transition from the dripping regime to the jetting regime. It has been studied that the angle between the disperse and continuous phase channels influences the droplet size, reaching a maximum when the angle is set at 90°.^[^ ^63^ ^]^ Our group used flow‐focusing microfluidics to produce photo‐crosslinking gelatin methacryloyl (GelMA) hydrogel microspheres in a previous study.^[^ ^64^ ^]^ GelMA precursor solution was broken down into droplets by the oil phase and solidified under UV irradiation. The microspheres made in this study were about 100 μm in size. In another study, Ahmed et al.^[^ ^65^ ^]^ fabricated a PDMS‐based microfluidic device to generate ion‐crosslinked alginate hydrogel microspheres. The CaCl_2_ solution encountered the oil phase at the flow‐focusing unit and turned into droplets, which were then crosslinked by alginate‐containing solution. Alginate microspheres with sizes less than 30 μm were obtained; however, the circularity was reduced. In another study, flow‐focusing geometry was constructed via assembly of glass capillaries and used to produce poly(acrylamide) (PAAm) microspheres.^[^ ^66^ ^]^

Headen et al.^[^ ^67^ ^]^ designed a parallel microfluidic device with a two‐layer structure to generate 4‐arm polyethylene glycol (PEG‐4MAL) microspheres loaded with human mesenchymal stem cells (hMSCs). This device was assembled through the bond of two photolithographically processed PDMS layers. The lower layer was patterned with a flow‐crossing geometry to generate hMSCs‐containing droplets and the upper layer formed microchannels communicating with the lower layer. As shown in Figure 2B, droplets generated in the lower layer were carried to the top layer, where they contoured the crosslinker solution and form solidified hydrogel microspheres. The advantage of the parallel flow‐focusing design was that it enabled the fabrication of highly uniformed microspheres with efficient cell encapsulation and high cell viability simultaneously. To increase the microsphere productivity, Kamperman et al.^[^ ^68^ ^]^ reported a more complex parallelized microfluidic device, which was made up of five radially arranged parallel flow‐focusing units, and each droplet generator had a three‐layer stack structure. The device was made from photo‐curable orange resin, fabricated via stereolithographic 3D printing technology. The high‐resolution fabrication of dextran (DEX) hydrogel microspheres from W/O emulsion templates was achieved in this study, and the mean microsphere diameter ranged from 150 to 1000 μm with coefficient variation (CV) < 10%. Besides, the production scale could be further enlarged by increasing the stack number or adding droplet generators on each stack.

As most hydrogel droplets are formed in a W/O system, the washing of the microspheres is always required to remove the adhered oil adhered, which is a tough work. Moreover, this is especially unfavorable for the microspheres loaded with cells since organic solvents are frequently used in the washing process, which can damage the cells. To overcome the shortcomings of W/O emulsion templates, Liu et al.^[^ ^69^ ^]^ proposed a flow‐focusing microfluidic approach for preparing microspheres in a water‐in‐water (W/W) system. The device was composed of three PDMS layers bonded together, where the top layer was patterned with inlet channels for introducing disperse and continuous phase, while flow‐focusing droplet generator and output channels were formed on the middle layer. Besides, the flow‐focusing unit was integrated with a pneumatic membrane value to form more stable and monodisperse droplets. The bottom layer was used as a substrate and all patterns were formed via lithography. The disperse and continuous phase consisted of two incompatible polymer solutions: dextran and PEG. The alginate disperse phase was prepared by mixing alginate into a DEX solution and a collecting bath containing CaCl_2_ solution was used to crosslink alginate droplets. Meanwhile, cells were allowed to load on the microspheres with good viability, via adding the cell suspension to the disperse phase, as shown in Figure 2C.

The main advantage of flow‐focusing junction is the significant enhancement on the shearing force, as the continuous phase squeezes the dispersed phase from two directions. Therefore, hydrogel droplets with extremely small sizes (< 10 μm) can be obtained using flow‐focusing microfluidic device, while maintaining a moderate CV.

### 3.2 Cross flow

Cross flow is characterized by its dispersed phase channel designed at an angle with the continuous phase channel. If this angle is equal to 90°, it is also called as T junction, which is the most popular type of cross‐flow geometry for generating droplets. Unlike the flow‐focusing geometry, at a low $N_{c}$ value, the disperse phase will not break up into droplets, but remains as a continuous fluid. When the $N_{c}$ value is high enough, the droplets are formed under the dominating viscous shear forces in the dripping regime. If $N_{c}$ continues to grow, it switches to the jetting regime. Xin et al.^[^ ^70^ ^]^ fabricated a microfluidic device that included a Y‐shape inlet and T‐junction droplet generator for manufacturing hydrogel microspheres crosslinked via thiol‐ene click chemistry. The device was made from PDMS, manufactured through standard soft lithography. The Y‐shape channel could not only enable the mixing of two different precursor solutions, including a four‐arm poly(ethylene glycol) (PEG)–norbornene and a thiol crosslinker, but also could control their flow properties. After flowing through the Y junction, the hybrid aqueous phase was squeezed by the continuous oil phase at T junction to form droplets, which were subsequently exposed to UV light to be solidified. The crosslinked hydrogel microspheres possessed high monodispersity, with an average diameter of 355 μm and CV of 2.5%.

**FIGURE 3:**
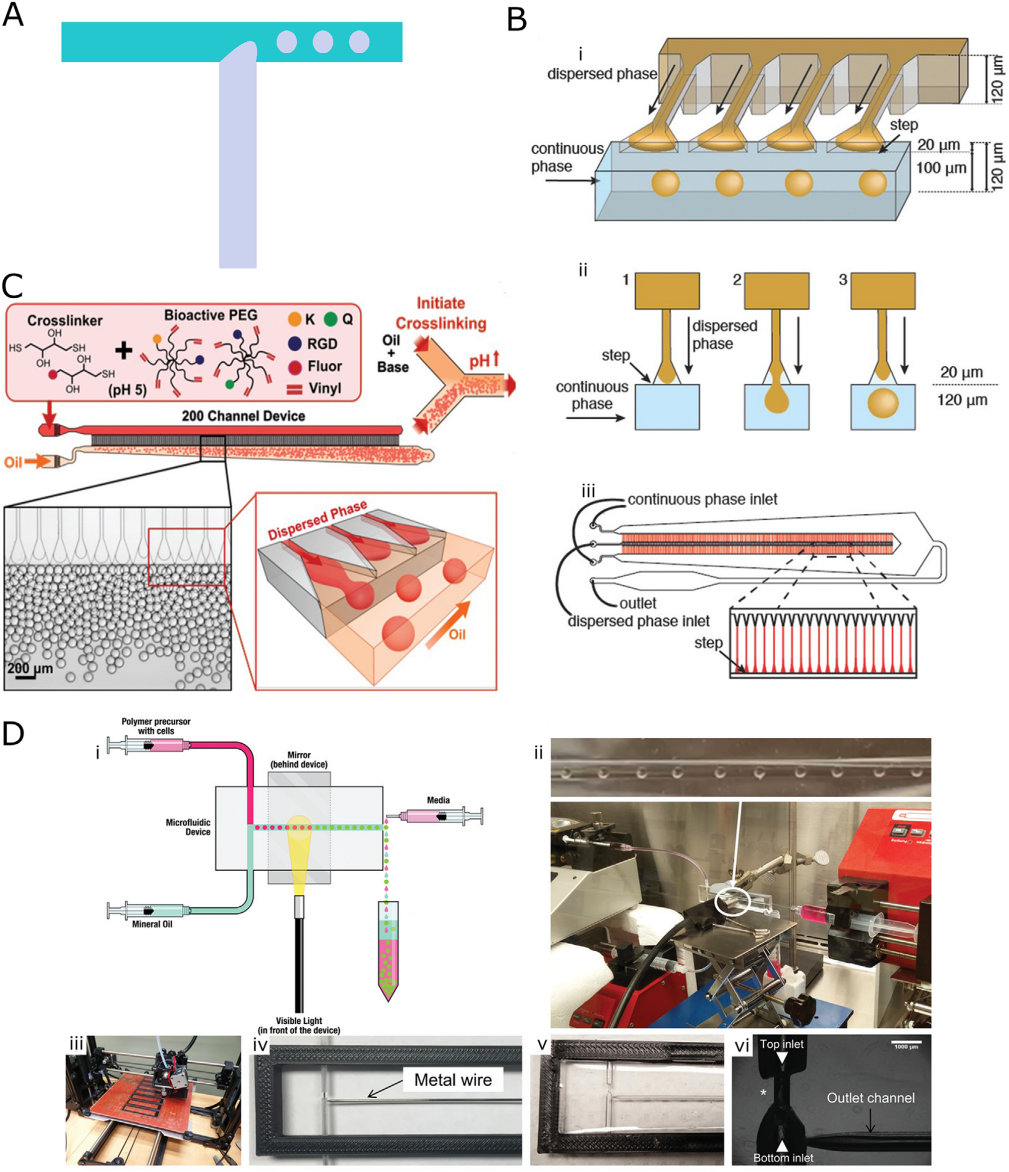
(A) Cross flow mechanism. (B) Design and working principle of step‐emulsification microfluidics: (i) Schematic of the step‐emulsification channel with four parallelized T‐junction droplet generator units. (ii) The process of step emulsification. (iii) Schematic of the complete microfluidic platform with 364 step‐emulsification units. Reproduced with permission.^[^ ^72^ ^]^ Copyright 2017, John Wiley & Sons. (C) Schematic of the parallelized step emulsification device with two‐step structure. Reproduced with permission.^[^ ^73^ ^]^ Copyright 2019, John Wiley & Sons. (D) Representation of a flexible microfluidic manufacturing strategy that used mold technique: (i) Schematic of the complete microfluidic platform. (ii) Droplet forming in microfluidic device. (iii–v) The fabricating process of the device. (vi) Droplet generator with T junction geometry. Reproduced with permission.^[^ ^74^ ^]^ Copyright 2019, John Wiley & Sons

Compared to the flow‐focusing geometry, cross flow significantly reduces the volume of the device since the continuous fluid comes only from the bottom channel (Figure 3A). Therefore, most parallel microfluidic devices for mass production are based on cross‐flow geometry. For example, Kim et al.^[^ ^71^ ^]^ designed a PDMS‐based 512‐channel microfluidic device with cross‐flow geometry, fabricated via conventional photolithography technique. A single channel was divided into two subordinate channels at every T junction, culminating in a tree‐like structure of 512 channels through nine segments. Having generated the ancestral droplets at the first junction, W/O emulsion flowed downstream into the next junction. The aqueous droplet was forced to split into smaller particles at each junction. This device was capable of producing chitosan microspheres with a mean diameter of less than 50 μm and a narrow size distribution at an emulsion flow rate of 10 ml/h, indicating that the multi‐T‐junction geometry had the potential to realize the large‐scale production of microspheres. Nevertheless, parallel cross flow microspheres with CV values higher than 7% cannot meet the demand of dispersity.

To improve the uniformity of microspheres while scaling up production, parallelized step emulsification platform has been proposed. Weitz et al.^[^ ^72^ ^]^ manufactured a glass‐based step emulsification microfluidic device using etching technique (Figure 3B), which included 364 linearly parallelized T‐junction units. When the disperse phase flowed through the ladder structure, droplets were formed due to the sudden expansion of the channel cross section. Since the disperse and continuous channels were not at the same level, the generation of droplets was not driven by the compression of the continuous phase relative to the dispersed phase, thus the flow rate made little impact on the droplet size. Poly(N‐isopropylacrylamide) (pNIPAm) hydrogel microspheres with an average diameter of 97 μm were generated, at a monodisperse emulsion flow rate of 25 ml/h (CV 4.6%). Furthermore, de Rutte et al.^[^ ^73^ ^]^ used soft lithography technique to fabricate a step emulsification device based on PDMS materials, as shown in Figure 3C. The microfluidic platform consisted of 200 disperse phase channels and one common continuous phase channel, which constituted 200 T‐junction structures. Unlike other parallelized devices, the mechanism of droplet generation was due to the sudden expansion of the channel height. At the same time, the geometry of channels presented a gradual expansion, which could improve the production rate and uniformity of droplets. The disperse phase consisted of poly(ethylene)glycol–vinyl sulfone (PEG–VS) and dithiol crosslinker, for acquiring hydrogel microspheres solidified via Michael addition reaction. On the other hand, this device was proved to achieve a higher production rate compared with parallelized flow‐focusing devices, with the emulsion throughputs of 30 ml/h and microsphere diameter of less than 100 μm (CV = 3%). It is noted that parallel cross‐flow device with a step structure can further reduce the size of the device, as all junctions are integrated on one main outlet channel thus parallelized step emulsification platform may be the solution for high‐volume production and industrialization of microfluidic microsphere.

Considering the complex manufacturing process of lithography, a more flexible and economical method for patterning T‐junction channels was proposed. Seeto et al.^[^ ^74^ ^]^ designed a microfluidic platform for rapid fabrication of cell‐laden microspheres based on T junction (Figure 3D). To produce droplet generator junction, a new strategy was proposed that avoided the involvement of expensive soft lithography. Teflon tubes and metal wires were used as channel molds, and then liquid PDMS was cast on the mold. After the removal of the mold from the cured PDMS, the channel pattern was formed. Tube‐ and wire‐based molds were able to generate channels with circular cross section while traditional soft photolithography methods could only produce rectangular cross‐sectional channels. In addition, to realize quick fabrication of photo‐crosslinking hydrogel microspheres, a visible light source was placed towards the outlet channel to solidify the precursor droplets. To verify the feasibility of the device, three types of photo‐crosslinking hydrogels were used to produce the microspheres, including PEG‐diacrylate (PEGDA), PEG‐fibrinogen (PF), and GelMA. Besides, diverse types of cells were mixed into the precursor solution to prepare cell‐laden microspheres with high cellular viability. However, as the junction was created from mold removal, the channel geometry and manufacturing accuracy were limited.

### 3.3 Co flow

**FIGURE 4:**
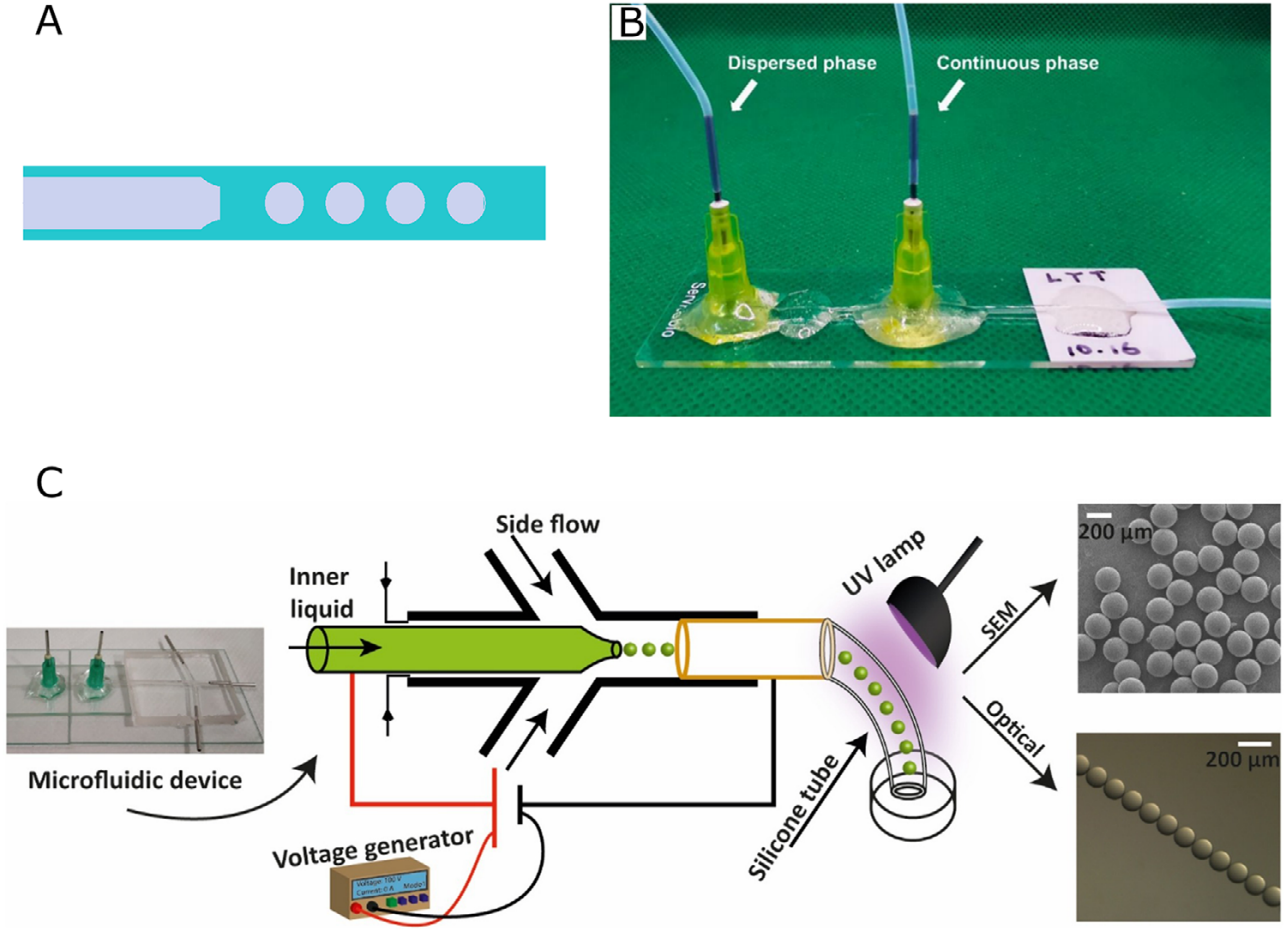
(A) Co flow mechanism. (B) Schematic of co‐flow device based on glass capillary for microsphere. The droplet generation geometry was formed by inserting the inner capillary into the outer capillary. Reproduced with permission.^[^ ^79^ ^]^ Copyright 2021, John Wiley & Sons. (C) Schematic of co‐flow microfluidic platform with electric field‐assistance for PEGDA microsphere. Reproduced with permission.^[^ ^80^ ^]^ Copyright 2020, Elsevier

Co flow refers to inserting the dispersed phase channel into the coaxial continuous phase channel so that the dispersed and continuous phase fluids can always flow in parallel through the channels (Figure 4A). As in flow‐focusing geometry, the dripping state occurs when the flow rates of both phases are low. In the dripping regime, the droplets are produced due to surface tension and set apart from the inner channel outlet under the drag force of the outer phase.^[^ ^20^ ^]^ The dripping‐to‐jetting transition can be achieved by varying the flow velocity of the dispersed or continuous phase and is explained by different dominanted forces. Viscous forces dominate at high continuous flow rates, while inertial effects become dominant by increasing the flow rate of the disperse phase.^[^ ^75^ ^]^ In the jetting regime, the inner fluid flows faster than the external fluid, causing the jet to expand and break up, followed by the formation of spherical droplets at the terminal of the jet. In a co‐flow device, the inner phase is totally wrapped by the outer phase, thus the droplets are formed in a three‐dimensional environment, in which the surface wettability of channels can be neglected.^[^ ^76^ ^]^ This channel structure can be easily constructed via glass capillary assembly strategy and various photo‐crosslinking hydrogel materials have been used for co‐flow microfluidic manufacturing.^[^ ^77^ ^]^ In previous studies, our group has used capillary‐based devices to prepare hydrogel microspheres made from GelMA or methacrylated hyaluronic acid and heparin (HAMA@HepMA).^[^ ^78^, ^79^ ^]^ The schematic graph of device is presented in Figure 4B. To assemble a co‐flow capillary device, briefly speaking, the inner and outer capillaries are connected and adhered to the same glass slide, followed by sealing process through bonding two needles. Capillary‐devices can be easily built even in general labs, making great progress in the promotion of microfluidic technology. For the production of hydrogel microspheres, the precursor solution is injected into the inner capillary while the outer capillary is filled with the oil phase to generate droplets.

Besides, some modifications have been made to enhance the functionality of glass capillary based microfluidics. Pullagura et al.^[^ ^80^ ^]^ (Figure 4C) applied electric fields on a co‐flow device to attain active generation and control of PEGDA hydrogel microspheres. The microfluidic platform was prepared by connecting a PDMS chip with glass capillaries, where glass capillary‐based co‐flow structure was used to generate a conventional W/O system. Two side channels and one central channel formed on the PDMS chip. Among them, the main function of the side channel was to provide extra oil phases if a high flow rate was needed. To attain electric manipulation, the inner glass capillary was assisted by a metallic needle, while another metal tube was inserted in the central channel as a ground electrode. A DC voltage generator was connected to the device to create electric fields. The size of the microspheres was affected by the applied voltage, ranging from 50 to 350 μm (CV < 3%).

Furthermore, Zhao et al.^[^ ^81^ ^]^ combined microfluidics with electrostatical technique to produce hollow alginate hydrogel microspheres. A typical coaxial capillary microfluidic device was used in which the inner capillary was filled with an air flow and an aqueous solution containing alginate was injected in the outer channel. Droplets containing the air core were generated under electrostatic field stimulation and collected in a water bath mixed with calcium ion to complete the crosslinking. Both the size and wall thickness of hollow microspheres were affected by the flow rate of two phases as well as the applied voltage, and the mean diameter of microspheres was between 200 and 800 μm in this research.

However, as the assembly of co‐flow capillary device is completely manually, it is challenging to precisely control the microfluidic parameter. In particular, the depth of the inserted inner capillary is inconsistent, and co‐flow devices of different batches are varied. In addition, although glass capillary is highly chemically resistant, resin glue is typically used to seal the capillaries. Therefore, the addition of organic solvents to the capillary devices is not permitted as it may cause damage.

### 3.4 Others

In addition to the basic droplet generator geometries mentioned above, in recent years, researchers have explored new droplet generation mechanisms and applied them to microfluidic devices, showing great promise for improving the production of hydrogel microspheres.

To enhance the producing efficiency, a distinctive channel design was developed by Akbari et al.^[^ ^82^ ^]^ Instead of using the popular strategy of parallel multi‐channels to achieve mass production of hydrogel droplets, the device was characterized by the customized channels with uniformly embedded micropillar array. The whole microfluidic system was built via soft lithography on PDMS. When the dispersed aqueous phase met the oil phase at the junction and flowed through the micropillars, the shear exerted by the oil phase would break up the aqueous phase into monodisperse droplets. Its maximum throughput could reach 3000 μl/h, which was higher than most parallelized cross‐flow devices, and hydrogel droplets of 8 μm in diameter (CV = 12%) were produced at a frequency of 3.1 MHz. Although micropillar device occupied less space than conventional parallelized geometries, the monodispersity of the droplets had reduced.

**FIGURE 5:**
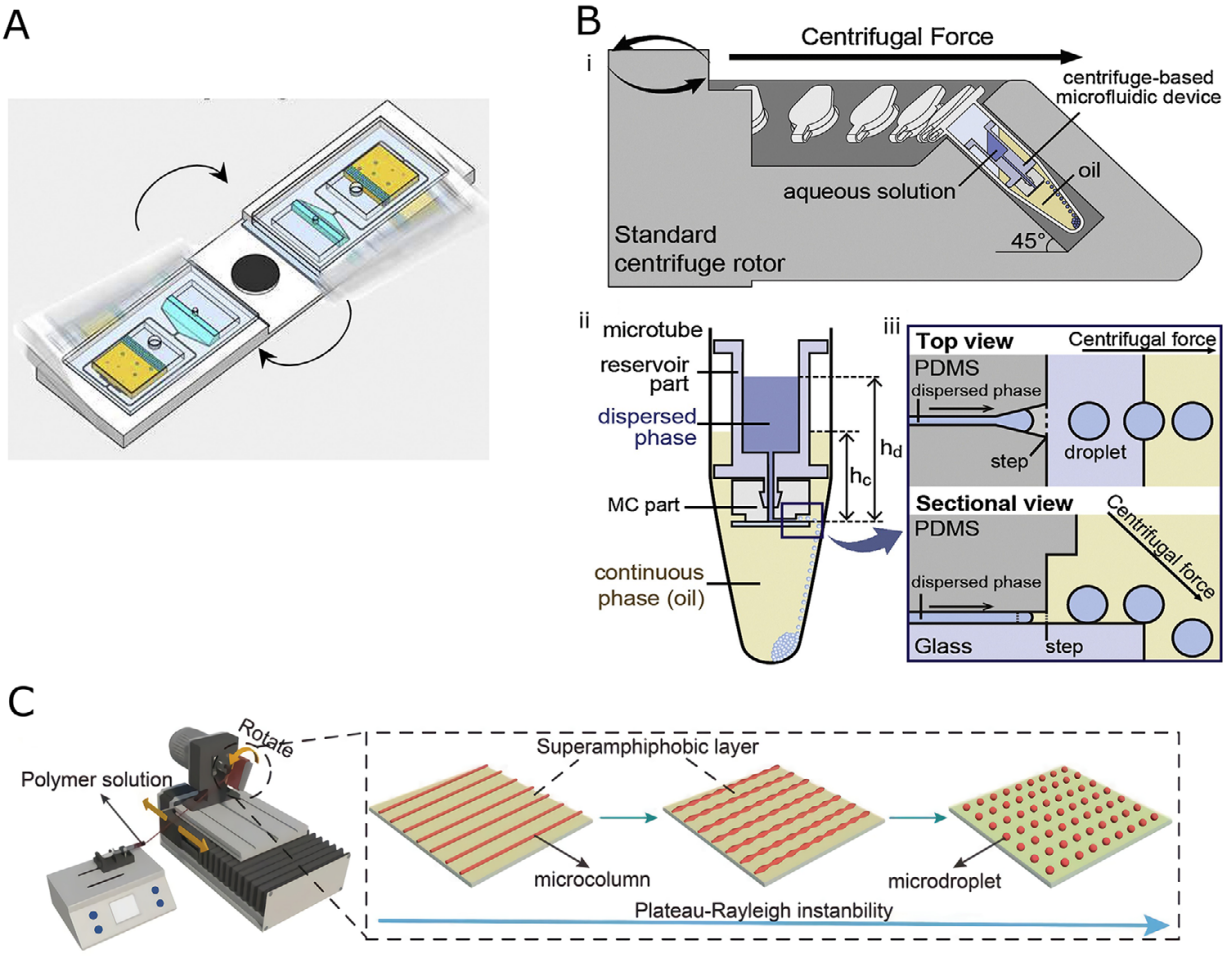
(A) Schematic of high‐throughput centrifugal droplet generator. Reproduced with permission.^[^ ^85^ ^]^ Copyright 2020, Elsevier. (B) Centrifugal microfluidic device: (i) Centrifugal microfluidics operated in a centrifuge rotor. (ii) Assembly diagram of the microtube. (iii) Schematic of droplet generation by centrifugal step emulsification. Reproduced with permission.^[^ ^86^ ^]^ Copyright 2019, Elsevier. (C) Schematic illustration of the customized microfluidic spin instrument and droplet forming from fluid instability. Reproduced with permission.^[^ ^89^ ^]^ Copyright 2021, John Wiley & Sons

High‐throughput centrifugal droplet microfluidics is another emerging approach for large‐scale production of hydrogel microspheres.^[^ ^83^ ^]^ As an off‐chip solution, centrifugal microfluidic devices can be easily assembled on conventional centrifuges with high scalability for large‐scale production of hydrogel microspheres.  In centrifugal microfluidic systems, the mechanism of droplet generation and detachment is due to an artificial gravity field produced by centrifugal force.^[^ ^84^ ^]^ Bond number ($B_{o}$) is used to describe the ratio of centrifugal force over interfacial tension, as follows:

(3) $$\;B_{o}=\frac{\Delta \rho gh^{2}}{\gamma }\;$$

where Δρ is the density difference between the dispersed and continuous phase, g denotes the gravitational force, h is the characterized height and γ indicates the interfacial tension between the two phases. Therefore, both centrifugal force and microchannel height can be used to predict the droplet generation behavior. Kim et al.^[^ ^85^ ^]^ used a centrifugal droplet microfluidic device to produce HAMA microspheres. The centrifugal microfluidic device consisted of an inlet and outlet chamber. The oil and aqueous phases filled the inlet chamber successively and oil flow would drive the aqueous through the microchannel that was connecting the inlet and outlet chambers during centrifugation, as shown in Figure 5A. In this way, highly uniform droplets were attained. To manufacture the device, a PDMS sheet was patterned with microchannels via soft lithography, while the inlet and outlet reservoirs were processed via mechanical cutting. Another PDMS substrate was performed hole‐drilling to form the inlet and outlet. These two PDMS and a glide glass were bonded successively using oxygen‐plasma oxidation. The water phase containing HAMA precursor solution and the oil phase were injected into the inlet and outlet, respectively. The microfluidic device was then assembled to the centrifuge and HAMA droplets could be harvested after centrifugation. Centrifugal microfluidic microspheres exhibited high monodispersity with a mean size of less than 100 μm. Moreover, the rotation condition had little impact on the size of microspheres, which proved the stability and feasibility of the device for hydrogel droplet production. Shin et al.^[^ ^86^ ^]^ (Figure 5B) developed a centrifugal step emulsification system that could be housed in common microtubes. The microfluidic device was mainly composed by two parts: the reservoir and gradually expanding microchannels. The reservoir was made from photoreactive resin, fabricated by stereolithography 3D printing technique, while the channel part was formed by soft lithography on PDMS. After fixing the device to the microtube, the continuous oil phase was poured into the microtube while disperse aqueous phase was injected to the reservoir. As the dispersed phase was pushed out through the channel by centrifugal force, hydrogel droplets were produced due to geometric confinement. PEG hydrogel microspheres were produced using this device, with the mean size of less than 100 μm and CV of 1.6%.

In addition to O/W emulsion template, W/W template is also available from centrifugal microfluidics. Takeshi et al.^[^ ^87^ ^]^ developed an oil‐free centrifugal microfluidic device. The droplets were produced using a customized microtube. A glass capillary with a sharp tip was assembled in the microtube and connected with the holder unit. The holder was used to separate the microtube into two compartments: one filled with hydrogel precursor and the other containing crosslinker solution. During the centrifugation, the hydrogel mixture was ejected from the tip of the capillary and formed monodisperse droplets, which would then fall into the crosslinker solution and finish the solidification. Alginate hydrogel microspheres with the mean size of about 250 μm (CV < 5%) were produced in this study.

Compared with conventional microfluidic techniques, the centrifugal microfluidics is capable of generating more uniform microspheres. That is because that the conventional on‐chip microfluidics requires propulsion pumps to inject the fluids and instrument errors may cause the change in flow rates. However, flow rate fluctuations can be neglected in centrifugal microfluidics since the fracture of the disperse phase is due to the hydraulic pressure difference during the centrifugation rather than the shear force exerted by the continuous phase. Another rotation‐based microfluidic strategy for droplet generation was developed by Tang et al.^[^ ^88^ ^]^ The whole platform could be mainly divided into three parts: direct‐current (DC) motor, conical frustum, and inlet needles. After the assembly of all the components, the frustum and the needles were immerged in a beaker filled with continuous oil phase liquid. The rotating motion of the frustum driven by the DC motor was able to lead the oil phase to attach to the surface of the frustum and to break the aqueous phase injected through the inlet needle, thereby generating hydrogel droplets. This droplet formation mechanism could be described by the low Reynolds of the microfluid, ensuring that the flow state was laminar even at maximum rotational speed. Droplets formed due to the breakup of the aqueous phase when the drag force exceeded the interfacial tension. Moreover, the size of the droplets was controllable through the modification of rotating velocity. PEGDA hydrogel droplets were prepared in this platform by mixing the PEGDA solution and photo‐crosslinker in the aqueous phase. After UV crosslinking, microspheres with the size ranging from 100 to 250 μm were obtained.

Song et al.^[^ ^89^ ^]^ (Figure 5C) reported a novel non‐chip approach for microsphere fabricating based on fluid instability template. A modified microfluidic spinning device was used to generate labile solution column that would further split into microspheres spontaneously. The system comprised of a spinning section and a rotating super‐amphiphobic layer. The amphiphobic layer was made by coating a thick hydrophobic silica layer on the sodium glass slide, which was then fixed to the rotator as a droplet collector. An intact precursor solution column stuck on the super‐amphiphobic layer would have infomogeneous curvature and shape the profile of droplet. This phenomenon was induced by the low surface energy of the superamphiphobic layer, which enlarged the interfacial tension at the liquid/solid interface. In this way, the fluid jet tended to break into droplets. After evaporating the solvent, microspheres were attained. Both the diameter of liquid column and the rotating speed could be adjusted to change the size of the microspheres, which could reach a minimum diameter of 5.3 μm while maintaining a good monodispersity (CV < 10%). Moreover, mass fabrication of microspheres was achieved, with a producing rate of 10^5^ spheres per second. Besides, this platform exhibited a high degree of versatility, being able to couple with various kinds of hydrogel precursor solutions, including both synthetic and natural hydrogels.

## 4 MICROFLUIDIC DEVICES FOR COMPOSITE HYDROGEL MICROSPHERE

Composite hydrogel microspheres integrating multiple functions have aroused extensive attention in the past decades. On the basis of the primary microfluidic technique for simple homogeneous hydrogel microspheres, nowadays, flexible design and modifications are being applied to conventional droplet microfluidic devices to manufacture more advanced and complex structured composite microspheres. In short, component hydrogel microspheres can be obtained from three emulsion templates, including composite single emulsion, double emulsion, and multi‐emulsion. Apart from the modification of classical microfluidic devices, innovative microfluidic strategies using novel droplet generation mechanism have also been proposed. Recent efforts in developing microfluidic devices for the formation of composite droplet emulsions are presented in this part.

### 4.1 Devices for compound single emulsion

Various modifications have been made on microfluidic devices to produce composite W/O or W/W single‐emulsion template with a wealth of features. To summarize briefly, these devices possess two or more disperse phase inlets and one continuous phase inlet for the generation of composite single emulsion.

Connecting multiple droplet generator junctions in series is a common method for producing composite single‐emulsion hydrogel microsphere. For example, Wang et al.^[^ ^90^ ^]^ developed a microfluidic chip for producing double‐core hydrogel microspheres. The chip consisted of one Y‐shape mixing unit and a cross flow droplet generator. In the mixing unit, two inner disperse channels were formed to generate double‐core structure and two other corresponding outer channels were used to produce shell component. In the downstream of the mixing unit, a flow focusing droplet generation unit was set. To manufacture the microfluidic chip, three PDMS sheets were used. The top PDMS was patterned with shell flow channels and inlets, while the other channels as well as mixing and droplet generator unit were fabricated on the middle PDMS. Soft lithography was used for all fabrication processes. The patterned top and middle PDMS sheets were then bonded to the bottom PDMS layer to finish the sealing. The aqueous core phase was loaded with Dex, while the shell flow contained PEG, alginate, and calcium disodium ethylene diaminetetracetate (Ca‐EDTA). The Dex flow and shell flow containing PEG could maintain a stable phase separation due to the high energy of interaction. Thus, after core fluid and shell fluid met and formed laminar flow, the W/W emulsion was squeezed by the continuous phase at flow focusing unit, where the inner aqueous phase turned into double‐core structure. Acetic acid (HAc) was added to the continuous phase so that C_a_^2+^ would release from Ca‐EDTA in an acidic environment and complete the crosslinking of the hydrogel microspheres. The mean diameter of the resulting microspheres was about 200 μm with CV < 5%.

**FIGURE 6:**
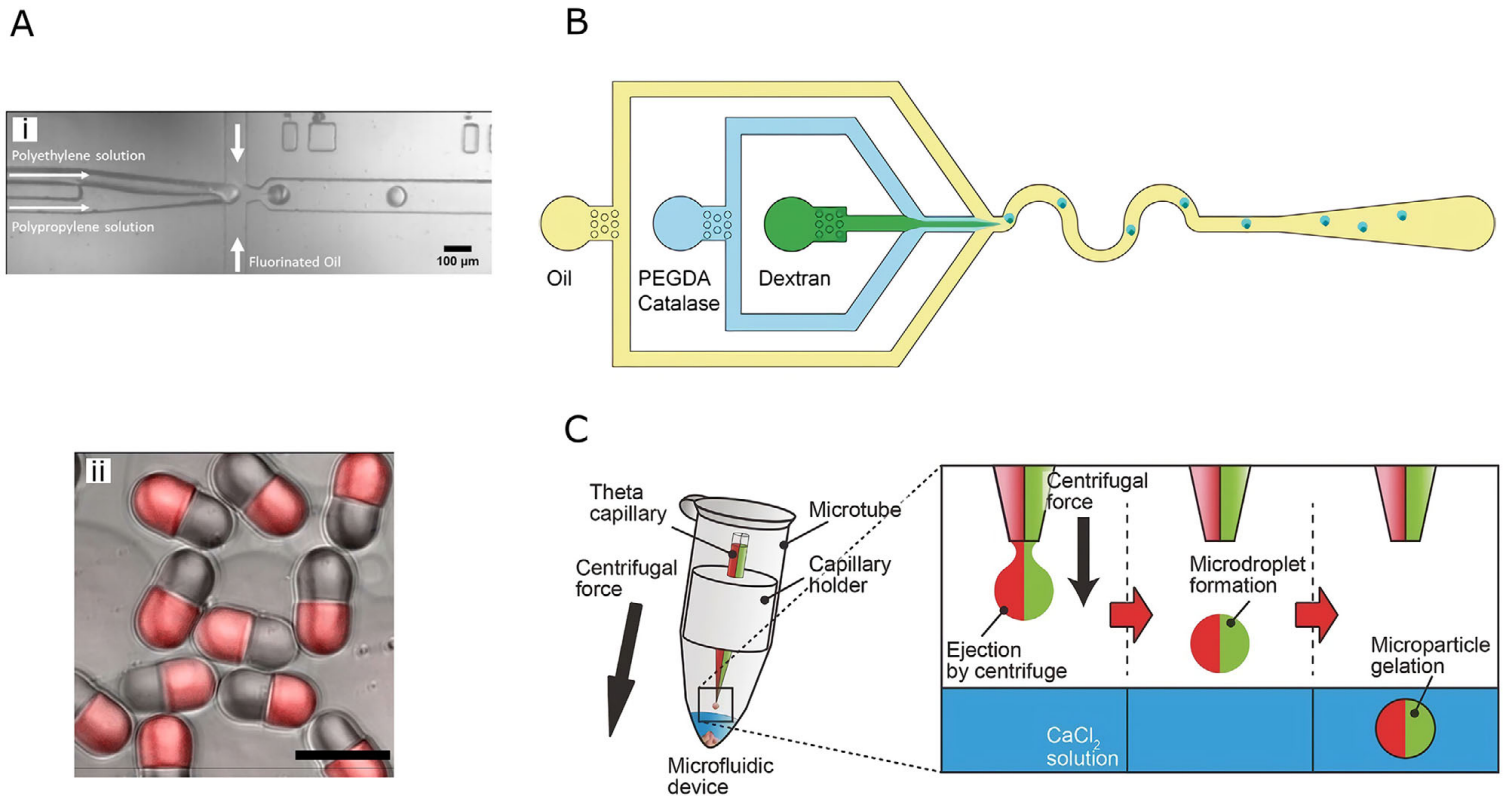
(A): (i) Optical image of the modified flow‐focusing device and formation of Janus microspheres. (ii) Synthesized amphiphilic Janus microspheres showing double compartments. Reproduced with permission.^[^ ^91^ ^]^ Copyright 2020, American Chemical Society. (B) Schematic of two‐step flow‐focusing microfluidic chip. Reproduced with permission.^[^ ^92^ ^]^ Copyright 2018, John Wiley & Sons. (C) Schematic of improved centrifuge‐based microfluidic device for generating composite microspheres composed of distinctive compartments. Reproduced with permission.^[^ ^93^ ^]^ Copyright 2017, John Wiley & Sons

Janus microspheres with two diverse components have also been produced via modifying classical microfluidic junctions. Haney et al.^[^ ^91^ ^]^ (Figure 6A) used a flow‐focusing chip to fabricate amphiphilic Janus droplets. Two parallel channels were patterned as disperse channels, which would converge at the flow focusing unit. All the geometries were formed on a PDMS sheet via soft lithography. Hydrophilic and hydrophobic polymer solutions were chosen as the disperse phase and remained in a laminar state at appropriate flow rates. Under laminar flow condition, the contact line between two disperse phases remained steady and parallel to the fluid direction. Thus, Janus droplets with two compartments were generated from two polymer solutions at equal flow rates. The average size of Janus microspheres produced in this work was about 56 μm in length and 28.5 μm in width, with excellent monodispersity (CV < 5%). In another study, a two‐step flow‐focusing microfluidics was developed for producing Janus droplets containing two separated phases.^[^ ^92^ ^]^ The device was also fabricated via photolithography on a PDMS substrate. As shown in Figure 6B, a two‐phase jet was formed at the first flow focusing unit, followed by emulsification of the oil phase into droplets at the second junction based on composite single‐emulsion template. PEGDA and dextran were chosen as aqueous solutions since they could ensure the laminar behavior. After UV crosslinking, PEGDA hydrogel microspheres were generated, with a small opening due to the diffusion of glucose in the hydrogel. The size and opening of the microspheres were controlled by changing flow rates, and the mean values were 19.8 and 8.2 μm, respectively.

However, as on‐chip strategy for compound emulsion is to simply connect multiple droplet generator junctions, they also face the similar shortcomings as most classic junctions, such as low productivity. Besides using conventional droplet generators, off‐chip microfluidic strategies have also been developed to produce composite microspheres. Yoshida et al.^[^ ^93^ ^]^ (Figure 6C) improved conventional centrifuge‐based microfluidic device to produce composite microspheres. Similar to standard centrifugal microfluidics, a capillary with sharp tip was fixed in the microtube to generate droplets. The characteristic of device was the tailored capillary that possessed two compartments. Two diverse ECM precursor solutions mixed with alginate filled the double‐chamber microtube and subsequently formed compartmentalized droplets under centrifugal force, which were crosslinked in the Ca^2+^ solution. Depending on the volume difference in each segment, the mixture can be ejected at different pressures in each segment so that anisotropic microspheres with inhomogeneous composition distribution can be obtained. The obtained alginate hydrogel microspheres had mean diameter of 118 μm and CV of 10.0%.

### 4.2 Devices for double emulsion

**FIGURE 7:**
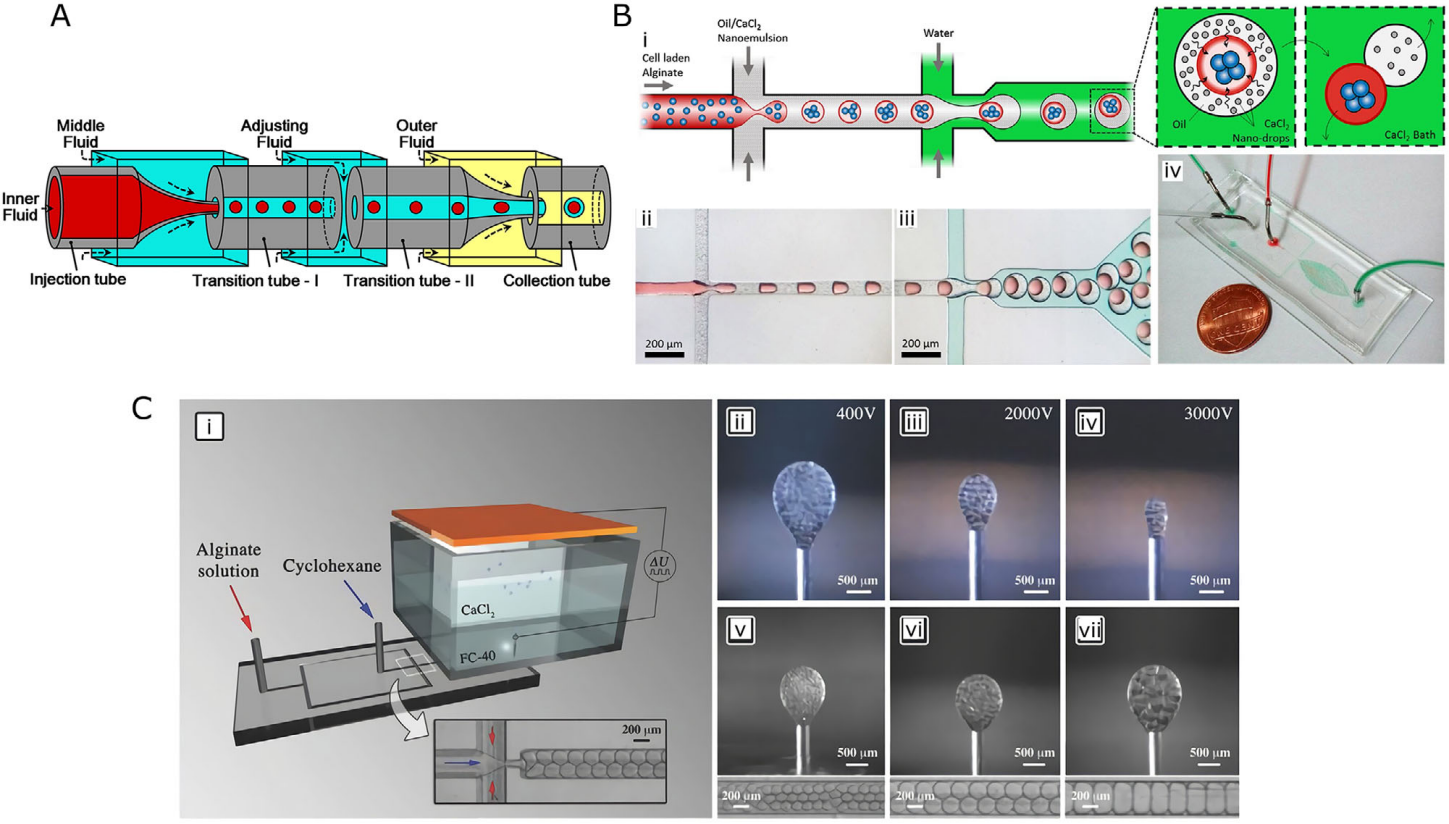
(A) Schematic illustration of glass capillary device for core/shell microsphere from O/W/O emulsion template. Reproduced with permission.^[^ ^95^ ^]^ Copyright 2020, Elsevier. (B) Generation of composite alginate microsphere using two‐step solidification in double emulsion. (i) Schematic of microfluidic device with two flow‐focusing units for cell‐laden O/W/O emulsion. (ii,iii) Optical image of droplet formation at two junctions. (iv) Profile display of microfluidic chip. Reproduced with permission.^[^ ^98^ ^]^ Copyright 2019, Elsevier. (C): (i) Schematics of the electric field‐assisted device for the manufacturing of porous microspheres from double emulsion. (ii–iv) Lateral light microscopy images show the formation of composite microspheres at different voltages. (v–vii) Lateral light microscopy images show the pore sizes of microspheres at different flow rates. Reproduced with permission.^[^ ^101^ ^]^ Copyright 2018, John Wiley & Sons

Multi‐stage emulsification system can be easily built on a microfluidic platform for preparing complex droplets. Hydrogel microspheres generated from double‐emulsion template possess core/shell structure, exhibiting higher complexity and richer functionality. A common method of forming double emulsions is based on coaxial capillary assembly. Conventionally, two tapered cylindrical capillaries are assembled coaxially in the square capillary, where the small tapered capillary is inserted into the untapered opening of another capillary. Capillary‐based devices have also been widely applied to produce W/O/W or O/W/O double‐emulsion template (Figure 7A).^[^ ^94^, ^95^ ^]^ In most cases, the two inner cylindrical capillaries and the outer capillary are sequentially connected in a line. The core phase and shell phase are injected through two inner capillaries, while the continuous phase flows through the interstices between the inner and outer capillaries. In addition, a binary W/O/W double‐emulsion template can be constructed by adding an inner capillary into the outer capillary.^[^ ^96^ ^]^

Alternatively, some researchers connected droplet generator units in series for the production of microspheres with a core/shell structure. For example, Liu et al.^[^ ^97^ ^]^ reported a strategy to construct core/shell hydrogel microspheres with highly efficient capability of cell encapsulation from W/W/O template. This platform consisted of two disperse phase inlets and one continuous phase inlet. The formation of droplets was attained through two coaxial flow‐focusing units where shell stream enfolded core stream at the first flow‐focusing unit, and the composite fluid was subsequently broken into droplets under the shear of the continuous oil phase at the second junction. In this research, cell culture medium solution was used as core fluid, while the alginate solution and CaCO_3_ particles were mixed to form the shell fluid. In particular, acetic acid was added to the oil phase to achieve in situ solidification of the hydrogel microspheres. That is because when it came into contact with the acidic oil in a low pH environment, Ca^2+^ ions would be released from the calcium complex, which would crosslink the hydrogel shell. As the flow rate changed, microspheres of diverse sizes were successfully fabricated, with the mean average size range from 200 to 600 μm (CV < 5%). Samandari et al.^[^ ^98^ ^]^ (Figure 7B) built a PDMS‐based platform consisting of two flow‐focusing units to generate W/O/W double‐emulsion template. The channel pattern was formed via conventional photo‐ and soft‐lithography approaches. Moreover, to improve the hydrophily of the channel surface, wettability patterning was performed, in which PVA was deposited on the plasma‐pretreated channels. Alginate solution loaded with cells was chosen as inner water phase, sheared by the oil phase containing Ca^2+^ at the first flow focusing unit to form droplets with the surface gelated. The W/O emulsion flowed downstream and formed composite O/W microspheres at the second flow focusing unit. At last, the microspheres were collected in an aqueous bath filled with CaCl_2_ to achieve complete gelatin. Previous methods of fabricating alginate hydrogel microspheres used a single emulsion template, where Ca^2+^ was added to the oil phase to crosslink the hydrogel droplets. However, this approach faced the drawbacks of unevenly crosslinking of the microspheres with non‐uniform size since the gelation process was slow. The two‐stage emulsion strategy was able to produce highly monodisperse microspheres, with the mean size of 72 μm (CV = 2.4%). Similar geometries have also been used to prepare W/W/O emulsions.^[^ ^99^ ^]^

Considering the demands of mass production, Nawar et al.^[^ ^100^ ^]^ reported multilayer parallelized microfluidics for double emulsions. Although parallelized microfluidic device has been widely applied for single‐emulsion droplets, it may not be suitable for double emulsion. That is because the surface modification of the channels must be spatially patterned in conventional parallelized microfluidics. For example, hydrophobic treatment is needed for generating W/O emulsion while hydrophilic modification is essential for the formation of O/W emulsion. However, it is quite difficult to accurately implement localized surface treatment on parallel devices. The advantage of the multi‐layer structure is that it allows independent channel treatment in each layer. In this study, the platform was established by laser cutting on four PMMA substrates. The top two layers were bonded and patterned flow‐focusing units with hydrophobic treatment. The second flow‐focusing junction was formed on the bottom two layers, which had undergone hydrophilic modification. Silicone acrylate hydrogel microspheres were produced from W/O/W double emulsion, with the average inner and outer sizes of 379.9 and 470.5 μm (CV = 5–7%), respectively.

In addition to combining multiple droplet generator geometries, Costantini et al.^[^ ^101^ ^]^ (Figure 7C) presented an electric field‐assisted microfluidic system for the preparation of composite porous microspheres based on double emulsion. This system consisted of a flow focusing unit and an electric field generator. Firstly, the alginate solution was injected into the continuous phase channel while the disperse channel was filled with cyclohexane. After cyclohexane phase was squeezed and formed into oil droplets, the O/W emulsion was then transmitted through a needle to a chamber that was filled with a thick layer of high‐density perfluorinated liquid (Fluorinert FC40) and an aqueous layer containing calcium chloride on top of the FC40. A pulsed voltage was applied to the liquid in the chamber to induce the fracture of a single emulsion and generated composite droplets based on O/W/O emulsion template. Then the droplets would ascend in the oil and solidify once they reached the Ca^2+^‐containing aqueous layer. When both the upward acting electric force $F_{E}$ and the buoyancy force $F_{C}$ exceeded the maximum capillary force $F_{C,\max}$, the droplet detached from the needle. As electric force $F_{E}$ was related to the properties of the electric field, which could be expressed as:

(4) $$\;F_{E}=\;\varepsilon U^{2}$$

where U is the external voltage and ε represents the constant coefficient. Therefore, the size of composite microspheres was affected by the field voltage and ranged from 200 to 800 μm (CV < 2%). By soaking the microspheres in dimethyl sulfoxide (DMSO), the oil droplets wrapped in the microspheres could be further removed to form a porous structure.

Recently, various innovative methods have emerged for double emulsions. Visser et al.^[^ ^102^ ^]^ built a novel in‐air microfluidic system that was free from traditional chip. In this system, only two nozzles were needed to assemble the microfluidic device, thus it dramatically reduced the fabrication cost compared with conventional on‐ship microfluidic devices. A customized nozzle was used in this system, assisted with a vibrating piezoelectric element. Under the reaction of the vibration component, the liquid jet was broken into homogeneous droplets. The in‐air droplets impacted onto the intact jet, which were then encapsulated and solidified by the jet. Due to the capillary force dominating the inertia, the droplets remained spherical, in other words, $N_{w}$ was no greater than 3. In particular, ν was described with the Equation (5):

(5) $$\nu \;=\nu_{D}\;\sin\theta$$

where $\nu_{D}$ is the ejection velocity of the disperse jet, and θ indicates the impact angle. Moreover, the difference in surface tension between the two jets ensured the successful encapsulation and curing of the droplets. A W/O/W double emulsion could be easily prepared by adding the surfactant‐containing fluorocarbon oil to the continuous jet. Moreover, it also allowed one‐step production of solidified hydrogel microspheres by mixing the crosslinker in the intact jet. Alginate core/shell hydrogel microspheres were generated by adding the alginate and Ca^2+^ solution to the disperse and continuous phase, respectively, the hydrogel microspheres had adjustable sizes by modifying the nozzle diameter and vibration frequency, ranging from 20 to 300 μm (CV < 5%). As a flexible microfluidic strategy, in‐air system could be extended to other areas. For instance, droplet‐based 3D printing was achieved by depositing solidified microspheres on a substrate.

Deng et al.^[^ ^103^ ^]^ purposed a microfiber‐templated strategy to produce hydrogel microspheres with diverse shapes. The microfiber‐based template was formed in two‐step co‐flow device. After the hydrogel precursor droplets were formed in a O/W emulsion at the first co‐flow unit, the single emulsion would be subsequently squeezed by the outer aqueous phase at the next co‐flow unit to generate microfibers. The oil‐core hydrogel microspheres were encapsulated into microfibers and could be collected by dissolving microfiber shell. Interestingly, the shape of microspheres varied according to the flow rates. With the increase of outer flow rate, the microfibers became thinner, leading to the elongation of the embedded droplets. Therefore, hydrogel microspheres with diverse morphologies could be obtained from the microfiber template, including sphericity, pear shape, and rod‐like shape.

### 4.3 Devices for multiple emulsion

**FIGURE 8:**
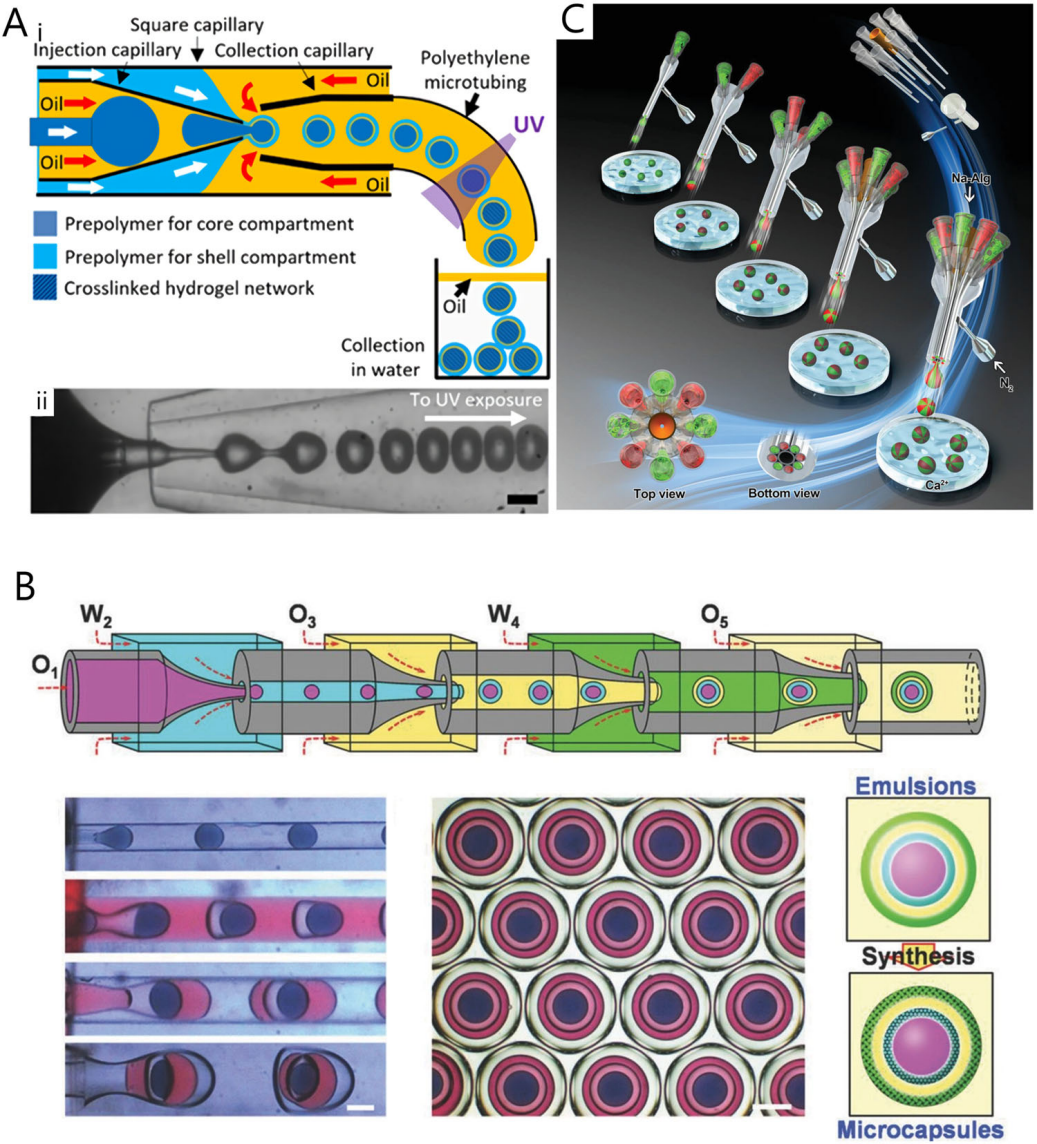
(A): (i) Schematic illustration of the glass capillary microfluidic device for triple emulsion. (ii) Bright‐field micrograph showing the formation of triple emulsion‐droplets. Reproduced with permission.^[^ ^104^ ^]^ Copyright 2021, Royal Society of Chemistry. (B) Schematic of glass‐capillary microfluidic device for generating O/W/O/W/O quadruple emulsion. Reproduced with permission.^[^ ^105^ ^]^ Copyright 2018, John Wiley & Sons. (C) Schematic diagram of the assembly of gas‐shear microfluidic system and multi‐compartmental microsphere production. Reproduced with permission.^[^ ^108^ ^]^ Copyright 2019, John Wiley & Sons

Through the assembly of multi‐stage capillary devices, multi‐emulsion template is produced for composite hydrogel microspheres. Quadruple‐capillary microfluidic is one of the most common methods for forming triple‐emulsion templates. Typically, the platform is built by assembling three circular capillaries into a square capillary. Three circular capillaries include collection capillary, injection capillary, and a smaller capillary inserted into the injection capillary. Liu et al.^[^ ^104^ ^]^ (Figure 8A) utilized quadruple‐capillary device to develop core‐shell hydrogel microspheres. Through adjusting the number of glass capillaries, microspheres with various shell structures could be obtained. Another method of constructing a multi‐stage capillary system is to connect co‐flow capillaries in series. Mou et al.^[^ ^105^ ^]^ developed a strategy to generate trojan‐horse‐like microspheres with capsule‐in‐capsule structures from multi‐emulsion template. As shown in Figure 8B, four circular capillaries were inserted into four square glass capillaries, respectively, for producing O/W/O/W/O quadruple emulsion. Poly(ethylene glycol) diacrylate (PEGDA), chitosan, and poly(*N*‐isopropylacrylamide) (PNIPAM) were added to the water phase to obtain composite microspheres with various kinds of hydrogel shells. All the microspheres exhibited high monodispersity, with the average size of about 400 μm and CV value lower than 4%.

Although glass capillaries can be easily assembled into multi‐stage devices to produce multi‐shell microspheres, they lack the ability to control the distribution of the components of the composite hydrogel microspheres. Gas‐shear microfluidics has emerged as a non‐chip strategy for preparing multiple‐emulsion microspheres with controllable compartment distribution.^[^ ^106^, ^107^ ^]^ Tang et al.^[^ ^108^ ^]^ developed a gas‐flow‐assisted co‐flow microfluidic platform, in which several liquid‐flow needles were inserted coaxially in a shell to build a spray ejector unit, connected with a gas holder, as shown in Figure 8C. The precursor droplets were generated under the shearing force of nitrogen gas and then collected in a bath containing crosslinker solution to form solidified hydrogel microspheres. The various fluids remained immiscible throughout the process and physically well separated after crosslinking. During the droplet generation, the force balance was given by Equation (6):

(6) $$\eta_{\mathrm{gas}}u_{\mathrm{gas}}d_{d}∼\gamma d_{t}$$

where $\eta_{\mathrm{gas}}$ denotes the gas viscosity, $u_{\mathrm{gas}}$ is the mean velocity of the gas, $d_{d}$ is the mean size of the droplet, $d_{t}$ is the diameter of the inner needle, and γ represents the surface tension. As the shear force exerted by the gas flow exceeded the surface tension, the droplet was separated from the liquid flow. Meanwhile, as an oil‐free method, gas‐shear microfluidics exhibited the potential in preparing cell‐laden microspheres since it avoided the washing operation. Moreover, via the modification of the needle number, microspheres containing up to eight compartments were fabricated, with size ranging from 55 to 1400 μm (CV < 2%).

## 5 OPTIMIZATION OF MICROFLUIDIC DEVICE

Although droplet microfluidics shows great potential in preparing single‐component and composite hydrogel microspheres, current microfluidic devices face many drawbacks that restrict their further application. For instance, the homogeneity of droplets varies from study to study, and the structural adjustment of microspheres is hard to implement. Moreover, complex surface modification processes need to be simplified and improved in efficiency. These issues require in‐depth optimization of microfluidic devices to improve their performance.

### 5.1 Enhanced droplet stability

In most cases, the disperse flow velocity is set very high to achieve a high yield of microspheres. As a result, the CV value increases drastically. This may be due to the phenomenon that when a mass of droplets enters the constricted entrance at the same time, they tend to deform or even break.^[^ ^109^ ^]^ On the other hand, large‐scale production of homogeneous droplets from highly viscous hydrogel solutions using conventional microfluidic devices is challenging. Thus, the content of hydrogels in the disperse phase is restricted, as a high concentration will lead to a high viscosity. To solve the above‐mentioned problems caused by the high flow velocity and viscosity of the hydrogel phase, several structural optimization strategies have been made on the microfluidic devices to improve the steadiness and homogeneity of droplet generation. Alison et al.^[^ ^110^ ^]^ reported a simple approach to avoid the simultaneous extrusion of droplets by fixing an obstacle in the outlet channel. When the obstacle was placed, droplets were forced to alternate and exit the outlet one at a time. The result showed that obstacle‐assisted microfluidics suppressed the breakup of droplets by three orders of magnitude.

**FIGURE 9:**
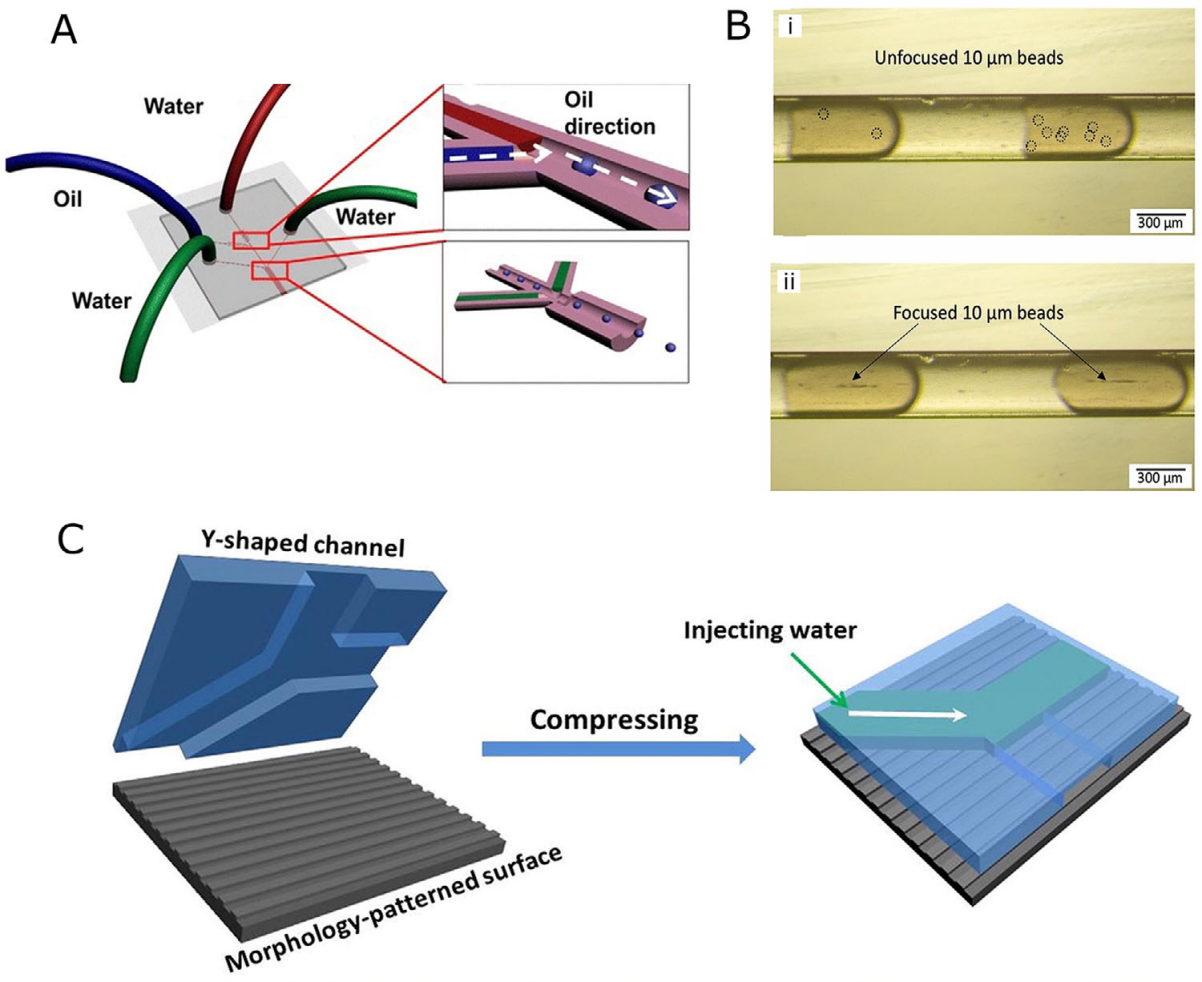
(A) Schematic of microfluidic channels with a zigzag structure. Reproduced with permission.^[^ ^113^ ^]^ Copyright 2018, Springer Nature. (B): (i) Photograph of randomly distributed nanoparticles embedded in hydrogel microspheres. (ii) Photograph of nanoparticles that are focused along the centerline under acoustic stimulation. Reproduced with permission.^[^ ^118^ ^]^ Copyright 2021, Springer Nature. (C) Schematic of fabricating channels with morphology‐patterned surfaces. Reproduced with permission.^[^ ^124^ ^]^ Copyright 2016, Royal Society of Chemistry

As the geometrical elements of the microchannels have great impact on droplet production, the stability of hydrogel microspheres can be improved via morphological adjustment of the microfluidic device. For instance, triangular junction channels in parallel microfluidic devices have been reported to improve droplet uniformity.^[^ ^111^ ^]^ For the disperse phase with very high viscosity, delayed retraction occurs at the standard rectangular junction, resulting in the decrease of droplet production rate and a wider size distribution. Compared with the rectangular channel, the triangular design is able to shorten the time for the dispersed phase to retract after droplet generation, thereby improving the size uniformity and production efficiency. A local modification of T‐junction channel has been proposed by Li et al.,^[^ ^112^ ^]^ where a rib was arranged in the downstream portion of the disperse phase channel. With the assistance of the rib, microfluidics exhibited the capability of generating more monodisperse droplets because the rib could enlarge the shear stress of the continuous phase and prevent the shear stress spreading across the channel wall. While most microfluidic channels are straight, nonlinear channels are designed with featuring zigzag structure to promote fluid stability (Figure 9A).^[^ ^113^ ^]^ In contrast with the common straight channel design, zigzag channel with several sharp corners exhibited higher robustness to the back pressure of the downstream fluid because the pressure drop between the inlets and the junction is enhanced.

### 5.2 Distribution control of cargos

Hydrogel microspheres are powerful tools for delivering cargos. However, in most cases, the structural optimization of microsphere carriers is neglected, limiting their application in accurate delivery. For example, as the cargos are easily mixed in the hydrogel solution, the distribution of the encapsulated cargos is always asymmetrical. When hydrogel microspheres are used as cell carriers, it is found that the cells distributed along the border of the hydrogel droplets tend to escape upon droplet crosslinking and during subsequent culturing.^[^ ^114^, ^115^ ^]^ Thus, the laden cells are expected to be focused on the center of the droplets to reduce or avoid cellular escape. In one study, microfluidic platform was placed on an orbital shaker that orbited at 1000 rpm during droplet crosslinking to achieve robust positioning of individual MSCs in the center of hydrogel microspheres.^[^ ^116^ ^]^ Nevertheless, the shaker and the microfluidic device are separate and cannot be integrated into one system. Furthermore, orbital shaking was limited to control single‐cell positioning and it was not clear whether it could be used for the localization of bulk cells. Recently, it has been discovered that acoustic fields can enable particle focusing within droplets and allow for integration into microfluidic devices.^[^ ^117^ ^]^ An acoustic focusing‐assisted microfluidic chip was developed by Fornell et al.^[^ ^118^ ^]^ for the positioning of bulk cells within hydrogel microspheres. Based on classical flow‐focusing geometry, this microfluidic device featured an exit channel assisted by a piezoelectric transducer to generate an acoustic standing wave field. The astrocytes encapsulated in the droplets were able to achieve oriented distribution under the action of acoustic force. And a UV light was fixed downstream to solidify the photo‐crosslinking hydrogel microspheres and immobilize the astrocytes. In this study, a combination of PEG and GelMA was chosen as the disperse phase due to the low viscosity and high biocompatibility. The acoustic manipulation showed no damage to astrocyte cells, which were successfully manipulated and concentrated along the centerline of the droplets under acoustic stimulation. In addition, acoustic focusing also provides the possibility of precise distribution control of the embedded particles within composite hydrogel microspheres. In another experiment, polystyrene beads were added to the hydrogel precursors, and precise localization was also achieved (Figure 9B). Since magnetic particle‐encapsulated composite microspheres are widely used as microrobots or micromotors because of their ability to acquire various motions in response to magnetic stimulation, controlling the distribution of encapsulated particles within the microspheres becomes particularly important because it determines the accuracy of the motion.^[^ ^106^ ^]^

### 5.3 Advanced surface modification of microchannel

The surface modification of microchannels is a critical fabrication process in manufacturing droplet microfluidic devices. Appropriate surface modification can improve fluid properties, ensuring stability of droplet generation in emulsion templates. For example, hydrophobic channel surfaces are necessary to produce W/O droplet emulsions while hydrophilic modifications are indispensable to generate stable O/W emulsion templates.^[^ ^119^, ^120^ ^]^ However, most of the modification methods are inefficient and cumbersome, especially for devices producing multi‐emulsion templates, as multiple surface modifications are required. In view of this, various efforts have been made to improve conventional surface modification technologies. For non‐assembled microfluidic devices, bonding and surface modification are two separate processes that are performed in sequence, and each process is time‐consuming. An ultrafast one‐step bonding and surface modification strategy was proposed by Su et al.^[^ ^121^ ^]^ for thermoplastic microfluidic devices. A one‐step solution was prepared by mixing 1H,1H,2H,2H‐perfluorooctyltrichlorosilane, acetone, and *n*‐pentane and spread on patterned polycarbonate (PC) substrates, and the modified PC substrates were subsequently bonded together via heated rolling. The rapid evaporation of *n*‐pentane during the hot rolling elevated acetone concentration, enabling fast and firm bonding of PC substrates. Moreover, FOTS molecules became entangled with PC polymer chains during heating and were trapped on the surface after solvent evaporation to make the microchannel hydrophobic. Stable water droplet generation in W/O emulsion was observed in hydrophobic‐treated thermoplastic microfluidic device, with a CV of < 1%.

In order to generate double or multiple emulsions, local surface modifications are essential to produce stable single emulsions at the different junctions. Li et al.^[^ ^122^ ^]^ proposed a locally hydrophobic modification strategy for producing W/O/W double emulsion in PMMA chips. The chip consisted of two flow focusing units, the first of which was used to generate W/O emulsions, while the second was hydrophobically modified via PDMS injection coating to generate double emulsions. This simple strategy also allowed local modification of other thermoplastic materials; however, modification based on thermal polymerization required precise flow control of the PDMS liquid flow. A less demanding surface treating method was proposed to deposit polyelectrolytes on hydrophobic PDMS microchannels using syringe‐vacuum‐induced flow segmentation to make them hydrophilic.^[^ ^123^ ^]^ A syringe was connected to the outlet channel to apply negative pressure so that polyelectrolyte solutions with opposite charges could be sequentially injected into the continuous channel. By manual pulling of the syringe, vacuum‐induced air‐polyelectrolyte segmented fluid only flowed through the continuous channel for selective deposition and local hydrophilic modification.

Recently, anisotropic wetting strategies that guide fluid properties have emerged as another powerful modification technique. Anisotropic wetting surfaces exhibit different hydrophilic properties in different directions, thus guiding the directional movement of liquid droplets. Wang et al.^[^ ^124^ ^]^ (Figure 9C) patterned the Si surface with a stripe geometry, and applied chemical vapor deposition to make the Si stripes hydrophobic. The hydrophobically modified channels showed anisotropic wettability due to the presence of different energy barrier levels along the direction perpendicular to the stripe. The anisotropic surface property is promising in controlling the flow behavior, such as the separation of two immiscible phases. For example, a hybrid structured microchannel with both hydrophobic and hydrophilic properties has been used for water‐oil separation.^[^ ^125^ ^]^ Therefore, anisotropic wetting modification may later be incorporated into the exit channel to enable rapid separation of emulsion templates and simplify the washing process of hydrogel microspheres.

## 6 CONCLUSIONS AND FUTURE DIRECTIONS

With the rapid evolution of micromachining and microtechnology, engineered microfluidic devices for hydrogel microspheres fabrication are gradually maturing. In the past, microfluidics was only allowed in well‐conditioned laboratories equipped with sophisticated hardware. The existence of capillary‐based microfluidics enables the production of micro‐materials even in ordinary laboratories, which greatly promotes the popularization and application of microfluidic technology. As hydrogels are prepared from the precursor solution, they can fully benefit from the progress of droplet microfluidic technology for the production of advanced micro‐materials.

In recent years, many strategies have been proposed to design microfluidic devices to generate complex multifunctional hydrogel microspheres. The current types of microfluidics as classified based on fabrication, have three categories: micro‐processing, modular assembly, and additive manufacturing. The determination of producing strategy depends on the property of the processed material and will further affect the subsequent microsphere formation. The key points of the droplet microfluidic device are channel and droplet generator junction designs. Microprocessing allows precise control of channel fabrication, while assembly and 3D printing provide a more flexible way to build microfluidic system. In addition to classical droplet generating units (flow focusing, cross flow, and coflow), various efforts are explored on optimization of microfluidic approaches and the new droplet formation mechanisms. Considering the remaining drawbacks of microfluidics and the requirements of future development, there are some vital topics needed to be further discussed and addressed.

1. Highly reusable devices are difficult to be produced for all types of microfluidics. The viscosity of the hydrogel precursor solution may lead to the clog of channels, which is tough to remove. In some cases, severely clogged microfluidic devices will be discarded. On the other hand, the majority of microfluidic channels face the risk of being polluted by the fluids. The contamination of microfluidic devices also restricts their reuse. Microfluidics requires more advanced and innovative materials to extend the service life of the devices.
2. Current microfluidics still fails to achieve one‐step formation of hydrogel microspheres. Most of hydrogel microspheres are attained by a two‐step method, in this case, an extra process is required to solidify the droplets or remove the oil phase. Actually, many microfluidic platforms for hydrogel microspheres come directly from droplet microfluidics, thus it lacks considerations of the production characteristics of hydrogel microspheres. Highly automated microfluidics integrated with solidification or phase‐separating unit is hoped to be developed in the future.
3. How to realize the industrialization of microfluidics remains challenging. Industrial applications require high throughput, excellent manufacturing yield, and acceptable producing cost. Although some improvements have been made to raise production rate, they are still far from the industrial demand. In addition, the balance between the size uniformity of microspheres and high output is hard to maintain. Obviously, the current efforts are only applicable to the laboratory environment, thus the potential in industrial applications needs to be further tapped.
4. Systematic research on the relations between microfluidic devices and hydrogel materials is lacking. In most studies, the newly developed platform is only restricted to the microsphere production of one kind of hydrogel. Whether it can be applied to a wider range of hydrogels remains unknown. After all, the property of hydrogel materials differs and one of the greatest challenges is the varying in viscosity for the hydrogels. The commonly used device may not be suitable for hydrogels with high‐viscosity, which are more likely to result in the channel blockage. Thus, the influence of device design on the formation of different types of hydrogel microspheres requires more in‐depth exploration.
5. The application of microfluidic hydrogel microspheres needs to be further broadened. The combination of microfluidics with other technologies is still rare. For example, the nozzle of the 3D printer has been applied for microfluidic modification in some researches so that microsphere composite scaffolds can be fabricated. The fusion of microfluidic hydrogel microspheres with other technologies is a wide topic. Therefore, a highly integrated platform that covers multiple functions is in need for preparing of multifunctional micro‐materials.

## CONFLICT OF INTEREST

The authors declare no conflicts of interest.
